# phylaGAN: data augmentation through conditional GANs and autoencoders for improving disease prediction accuracy using microbiome data

**DOI:** 10.1093/bioinformatics/btae161

**Published:** 2024-04-03

**Authors:** Divya Sharma, Wendy Lou, Wei Xu

**Affiliations:** Biostatistics Department, Princess Margaret Cancer Center, University Health Network, Toronto, ON, M5G2C4, Canada; Division of Biostatistics, Dalla Lana School of Public Health, University of Toronto, Toronto, ON, M5T3M7, Canada; Division of Biostatistics, Dalla Lana School of Public Health, University of Toronto, Toronto, ON, M5T3M7, Canada; Biostatistics Department, Princess Margaret Cancer Center, University Health Network, Toronto, ON, M5G2C4, Canada; Division of Biostatistics, Dalla Lana School of Public Health, University of Toronto, Toronto, ON, M5T3M7, Canada

## Abstract

**Motivation:**

Research is improving our understanding of how the microbiome interacts with the human body and its impact on human health. Existing machine learning methods have shown great potential in discriminating healthy from diseased microbiome states. However, Machine Learning based prediction using microbiome data has challenges such as, small sample size, imbalance between cases and controls and high cost of collecting large number of samples. To address these challenges, we propose a deep learning framework *phylaGAN* to augment the existing datasets with generated microbiome data using a combination of conditional generative adversarial network (C-GAN) and autoencoder. Conditional generative adversarial networks train two models against each other to compute larger simulated datasets that are representative of the original dataset. Autoencoder maps the original and the generated samples onto a common subspace to make the prediction more accurate.

**Results:**

Extensive evaluation and predictive analysis was conducted on two datasets, T2D study and Cirrhosis study showing an improvement in mean AUC using data augmentation by 11% and 5% respectively. External validation on a cohort classifying between obese and lean subjects, with a smaller sample size provided an improvement in mean AUC close to 32% when augmented through *phylaGAN* as compared to using the original cohort. Our findings not only indicate that the generative adversarial networks can create samples that mimic the original data across various diversity metrics, but also highlight the potential of enhancing disease prediction through machine learning models trained on synthetic data.

**Availability and implementation:**

https://github.com/divya031090/phylaGAN.

## 1 Introduction

The microbiome is defined as a community of microorganisms that inhabit a particular body site or environment (Ursell *et al.* 2012). Microbiome is dynamic in nature and is influenced by microorganisms and their interactions, their interplay with the host and the surrounding environment, and further by constant co-evolution (Berg *et al.* 2020).

Recently, machine learning (ML) models have been advocated for a data-driven approach for the prediction of the host phenotypes (Pasolli *et al.* 2016, LaPierre *et al.* 2019, Sharma *et al.* 2020, McCoubrey *et al.* 2021, Sharma and Xu 2021). However, persistent challenges such as the relatively small sample size in microbiome datasets and imbalance between the number of cases and controls hamper the performance of predictive ML models using microbiome data as input. It is often the case that microbiome datasets have a far greater number of features than the number of samples, which can quickly lead to the overfitting of models.

In recent times, Generative Adversarial Networks (GANs) have gained popularity for synthesizing new data from existing samples. GANs involve two neural networks competing against each other in an adversarial fashion in order to learn a generative model in a nonparametric data-driven approach (Goodfellow 2016). GAN models have shown success in multiple domains including the generation of medical images (Frid-Adar *et al.* 2018) and single cell RNA-Seq gene expression profiles (Ghahramani *et al.* 2018). In addition, synthetic datasets generated using GAN models have shown to be able to boost performance of prediction based tasks through data augmentation (Che *et al.* 2017, Choi *et al.* 2023). A recent study has also explored the behavior of GAN models for generating better microbiome data as compared to other simulation techniques (Rong *et al.* 2021). However, the study does not fully explore improvement in prediction models using the generated data.

Autoencoders are another form of ML models which have been used for effective representation of microbiome profiles (Oh and Zhang 2020, 2021). Taking motivation from the capabilities of GANs and autoencoders, in this paper, we use a combination of both these models for effective representation of the augmented microbiome dataset. This can be further used for enhancing the performance of microbiome based disease prediction tasks. We propose the use of a variation of standard GAN models called Conditional GAN (C-GAN) (Dai *et al.* 2017). C-GANs incorporate output label (case or control) of the samples into the model to allow the generation of samples from different distributions leading to improvement from standard GAN models (Mirza and Osindero 2014). We then proceed toward using autoencoders for projecting both the original and the C-GAN generated data onto a common subspace (Sharma *et al.* 2019) to enhance the prediction performance.

Research supports that simulating microbiome abundances with high similarity to the real data has its own challenges. Microbiome properties such as abundance demonstrates characteristics of sparsity, having large variances compared to means, and an inherent hierarchical structure owing to the phylogenetic and taxonomical relationship between the Operational Taxonomic Units (OTUs)(Li 2015). In addition, the microbiome data consists of interactions between OTUs forming complex negative, positive as well as, nonlinear taxa–taxa relationships (Faust *et al.* 2012, Layeghifard *et al.* 2017, Sharma *et al.* 2020). As per the current research, a data generative method that can capture microbiome properties accurately such that the generated microbiome data can be directly used for augmentation and enhancement of the predictive performance is missing in the literature.

**Figure 1.**
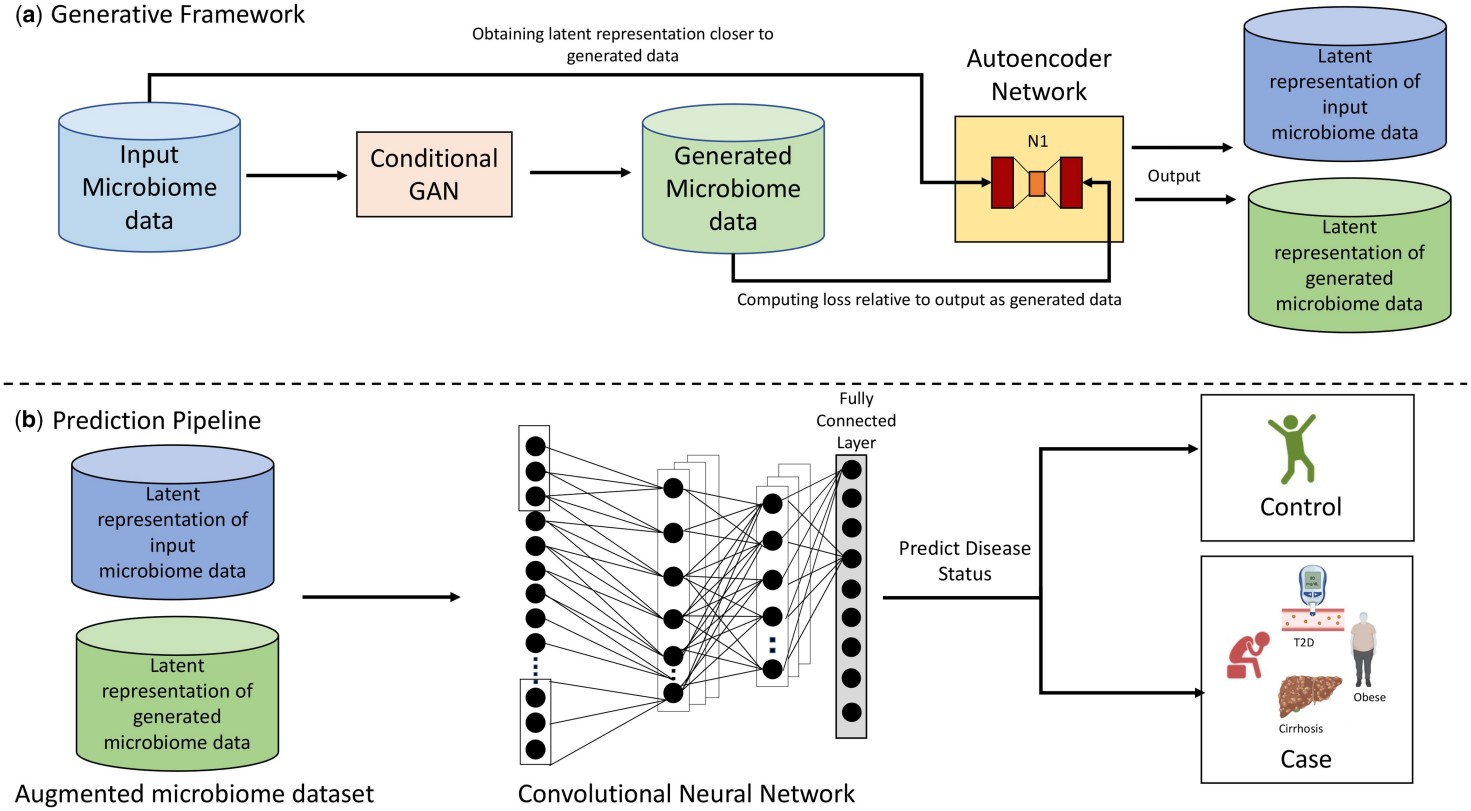
Overall Framework of *phylaGAN* for the integrated generative and predictive framework. (a) The generative framework with a combination of Conditional GAN and Autoencoder network to map the generated and original microbiome samples onto a common subspace for extracting the latent representation to act as an input for the prediction pipeline. (b) Prediction pipeline with the stratified Convolutional Neural Network (CNN) to extract features from the augmented microbiome dataset to accurately predict disease status using ensemble of CNNs.

To overcome these challenges in simulating microbiome datasets we take advantage of an end-to-end deep learning methodology to generate microbiome samples similar to the original data as well as utilize them for prediction of disease status. We propose a framework *phylaGAN* as shown in Fig. 1. The key points of novelty of our methodology are: (i) two-step generative pipeline using a combination of C-GAN model and autoencoders that can sample microbiome data from different conditions and provide synthetic data representative of the true data and project it onto a common subspace for disease prediction, (ii) end to end pipeline for prediction along with generation, wherein, even if the generated microbiome samples are not entirely similar to the original data, our autoencoder framework is able to fill in the gap through latent representation of the microbiome data for efficient prediction, and (iii) through transfer learning approach we show that the models trained on larger datasets can be used on small datasets as well, mitigating the lin larger datasets can be used on small datasets as well, mitigating the limitation of GAN networks to underperform with small datasets. Through this paper we show that the generated data is not only similar to the original data with respect to diversity metrics, but also that the data augmentation can lead to significant improvement in performance of ML models.

## 2 Materials and methods

### 2.1 Proposed framework

The methodology framework is divided into two pipelines: (i) Generative pipeline: we adopt a two-step framework where, as shown in Fig. 1a, we first generate the microbiome data through a Conditional GAN framework and further in the second step use an autoencoder network which projects the original and generated data to a similar space making it suitable for the prediction task. (ii) Prediction pipeline: we then proceed toward the prediction pipeline (Fig. 1b), where we stratify our input OTU data into various clusters based on their phylum information (Sharma *et al.* 2020). Further, as ensemble learning is effective (Ju *et al.* 2018), we use an ensemble of CNNs over the stratified clusters containing OTUs sharing the same phylum. The rationale is that OTUs after the phylum level division share a common parent phylum and hence, some association with each other. Moreover, to introduce spatial relationship in the input OTUs for the Convolutional Neural Networks (CNNs) to capture, we order the OTUs on the basis of correlation with each other to predict disease status accurately.

### 2.2 Conditional generative adversarial networks

**Figure 2.**
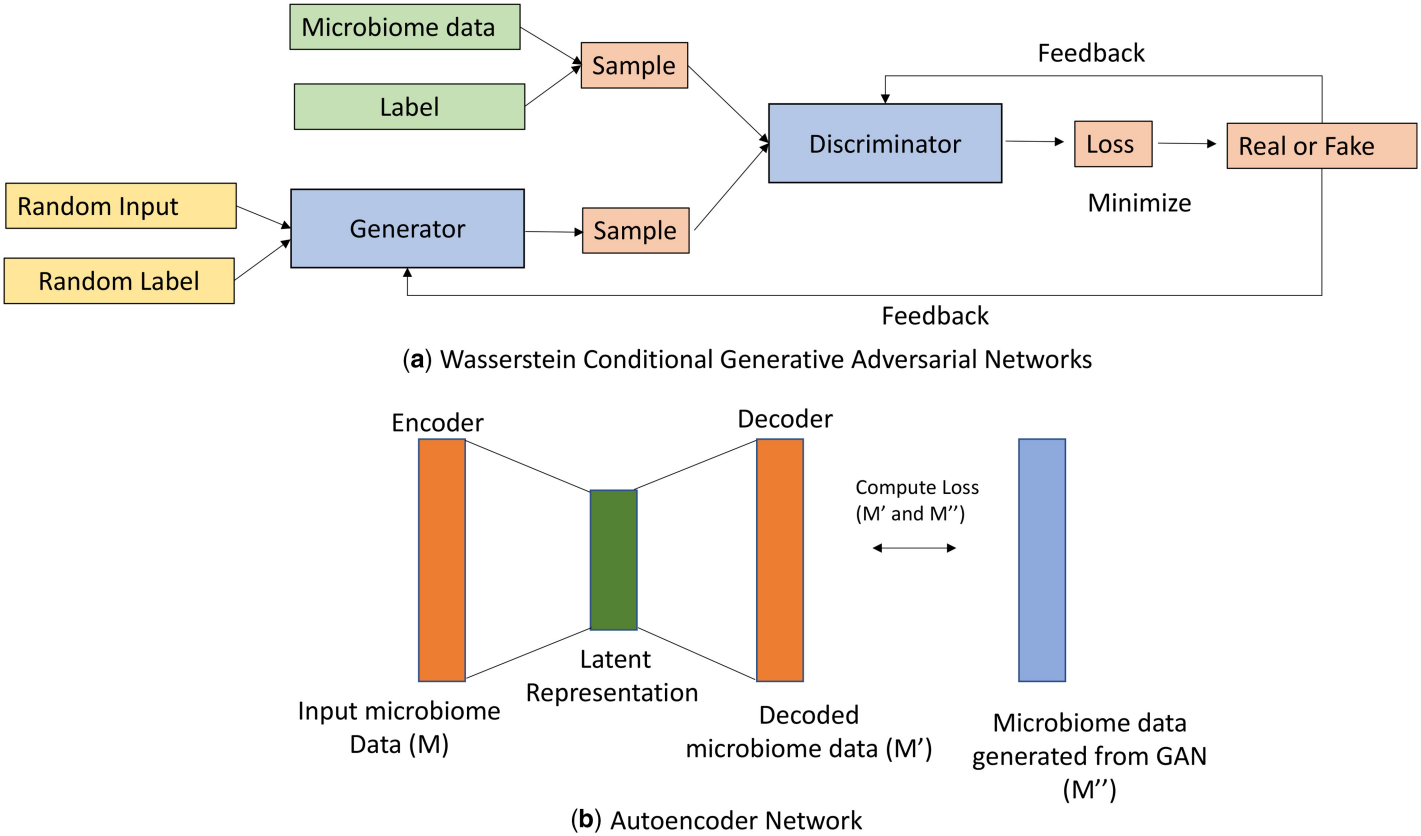
Generative framework. (a) Conditional Generative Adversarial Network representing generator and discriminator blocks which work together to produce samples as close to the original dataset. C-GAN has an additional input as the label of the microbiome sample to distinguish between cases and controls while producing new data. (b) Layerwise depiction of the autoencoder network. Note that the output error is altered to compute loss between decoded microbiome data and the generated samples to project both generated and input microbiome data onto a common subspace.

The C-GAN framework has two parts, a generator and a discriminator where the input data also incorporates the labels of the samples to help distinguish between cases and controls while generating new samples. Let, the original microbiome domain be denoted as *M*. An *i*th microbiome sample is denoted as $f_{M}^{i}$, where, i=1,2,…,m. Here, *m* is the number of samples in the original dataset. To convert a microbiome sample to its corresponding generated version, we introduce a mapping function G(.). The task of G(.) is to take a microbiome sample from the original dataset and convert it to the generated sample (${\bar{f}}_{M}^{i}$), which closely resembles the original sample. This process is denoted in Fig. 2a. To compare between the actual and the desired distributions, we have introduced an adversarial discriminator D(.). The task of D(.), is to check how good the mapping is from a given microbiome sample to the generated one. During the training phase, examples from the original dataset are given to the network to learn mapping function G(.). Transform G(.) aims to fool a discriminator which is simultaneously learning to discriminate between real and synthetic data in the microbiome domain.

Let the probability distribution of a sample $f_{M}\in M$ be denoted as $p(f_{M})$ and *class* be the class label (case or control), then the adversarial loss function for mapping a microbiome to its generated sample (${\bar{f}}_{M}$) can be written as:


$$\begin{array}{c} min_{G}max_{D}L\mathrm{oss}(G,D)=E_{f_{M}\to p_{(f_{M}})}[\mathrm{logD}(f_{M}|\mathrm{class})]\\ +E_{{\bar{f}}_{M}\to p_{({\bar{f}}_{M}})}[\log(1-D(G({\bar{f}}_{M}|\mathrm{class}))] \end{array}$$

In above equation, two operations are taking place. Here, the discriminator term D(.), tries to maximize the objective function by distinguishing between the generated microbiome samples and the original microbiome data. Whereas, the generator function G(.) tries to minimize the objective against the adversary and synthesizes microbiome samples as close to the original data.

As shown in Fig. 4, the generated target domain sample using G(.), is close to that of the source domain. The learned mapping functions are able to map to any random permutation of microbiome data. However, it does not ensure that for a given microbiome sample $f_{M}^{i}$, a corresponding ${\bar{f}}_{M}^{i}$ with exactly the same properties is obtained. Hence, to bridge the gap between the original and generated samples we use an additional autoencoder network as described in the next subsection.

### 2.3 Autoencoder for latent embedding of generated and original microbiome data

As observed in Fig. 4, function *G* generates a microbiome distribution corresponding to the original microbiome sample *M* that is approximately similar to how a sample would be in the real dataset. However, the approximation is still a little far from the exact actual representation (refer Fig. 4a where abundance varies a bit). In turn, we observed that using the generated sample directly in the augmented dataset along side the original microbiome data, the AUC values obtained were as low as 0.63 on the Cirrhosis dataset (Qin *et al.* 2012) due to the gap between the generated and original samples. Therefore, to overcome these shortcomings, we propose the use of autoencoders. This helps in mapping the original microbiome and generated microbiome data onto a common space for proper augmentation purpose.

An autoencoder neural network is an unsupervised learning algorithm that applies backpropagation and sets the target values to be equal to the inputs, i.e. it uses $y^{i}=x^{i}$. The autoencoders in our framework are not being used to generate output microbiome data similar to the input data at the output layer, rather the use of autoencoders is proposed to learn a common subspace representation of two similar looking domains. Therefore, in our case, additional output data (generated using Conditional GAN) corresponding to the input data is used for training the autoencoders (Fig. 2b). This additional output helps to learn latent representation of both input and output domains. This intermediate latent representation acts as a common subspace representation in our case. The novelty lies in the fact that, unlike traditional autoencoders, which try to generate an output similar to the input fed to them by computing the loss between the input and the target output and updating the weights, the training process of the proposed autoencoder is slightly modified. In the proposed case, the aim is to minimize the difference between the decoder generated output and provided output (generated through C-GAN). As shown in Fig. 2b, an autoencoder consists of two parts, the encoder and the decoder, which can be represented as E and D respectively. Where, E:i→z and $D:z\to \hat{i}$, and z is the compressed representation of the input. Note that during training the target has been set as the microbiome data generated, so that the autoencoder learns to map both domains.

Further, the squared loss function to minimize the reconstruction error to train the network is defined as follows:


(1) $$\begin{array}{c} L(\hat{i},i^{\prime })=||\hat{i}-i^{\prime }|{|}^{2} \end{array}$$

Note that, $\hat{i}$ is the reconstructed sample from the trained autoencoder corresponding to the input. The distinction of our autoencoder network from a simple autoencoder is that the loss is calculated between the GAN generated sample and the reconstructed sample. The parameters for the autoencoder network are listed in Supplementary Table S1. After the network is fully trained, features from the last layer of the encoder are extracted for both the original microbiome data and generated microbiome data from the *phylaGAN* dataset. This helps to obtain a compressed feature vector for both datasets, which is further used for prediction task when augmented together. This feature extraction helps in mapping both original and generated microbiome data to a common latent domain using which the prediction task can be carried out.

#### 2.3.1 Feature extraction through stratified CNN

**Figure 3.**
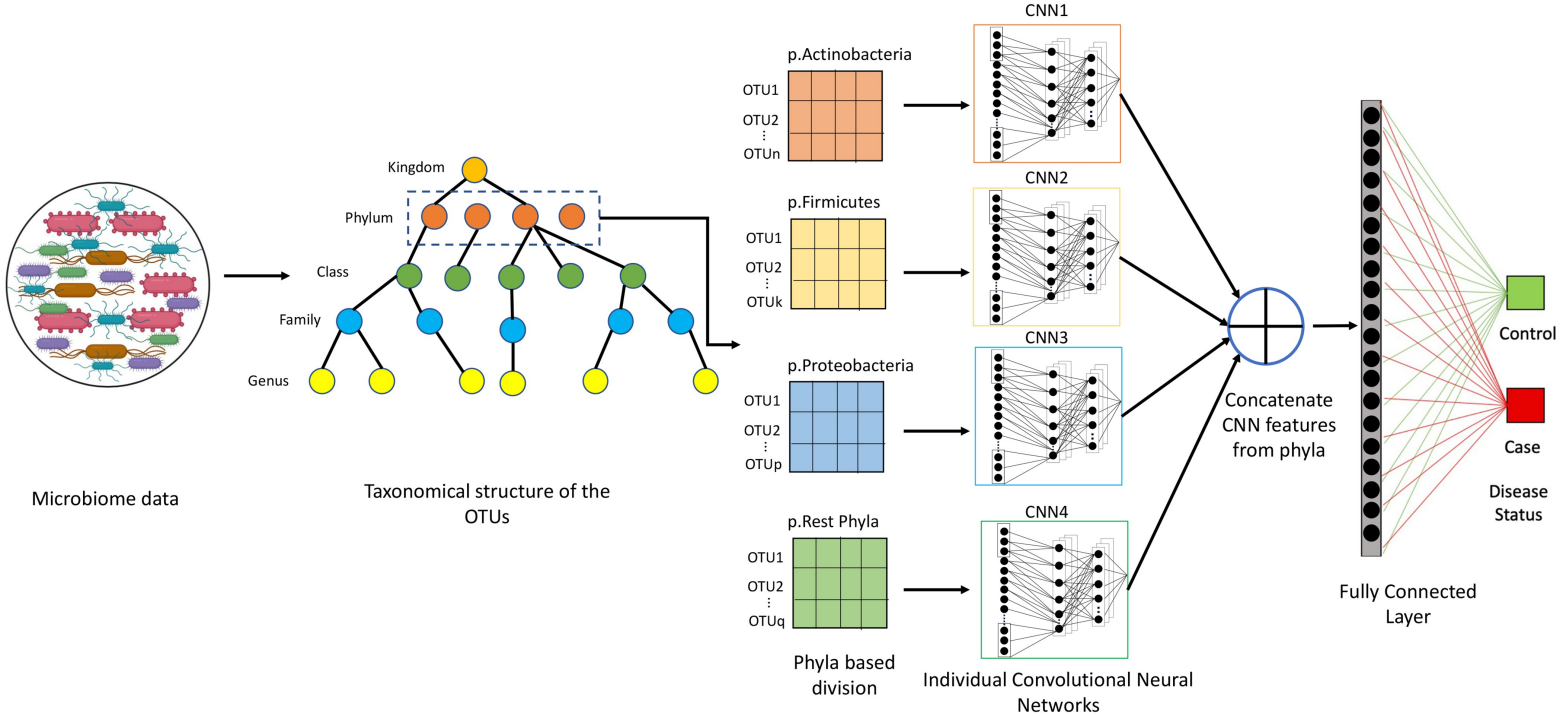
Detailed illustration of the $\mathrm{taxoN}N_{\mathrm{corr}}$ framework for phylum based stratification and ensemble learning of CNNs for disease prediction from the microbiome data. Based on phyla containing the majority OTUs, input microbiome data is clustered into four main clusters and provided as an input individually to the four neural networks (CNN1, CNN2, CNN3, and CNN4). Later the features extracted are flattened and stacked during the concatenation step to further lead to prediction of disease outcome through a fully connected layer.

As shown in Fig. 3, to extract features efficiently from OTU data, we use a CNN network suited for the OTU data structure. Let there be ‘n’ subjects in the whole study. The OTU data for *i*th subject (where, i∈ n), is presented in a 1D vector format to the network, as, $OTU_{i^{th}s\mathrm{ubject}}=\{o_{1},o_{2}\ldots .,o_{N}\}$, where, N is the total number of OTUs in a subject. In place of using unstructured OTU data, without looking into spatial relationships in the data, we use a stratified approach, where we first divide OTU data into phyla groups. The phyla groups are chosen based on phylum containing majority of OTUs. Further, ensemble of convolutional neural networks is applied to each phyla group to extract features. Herein, two aspects of OTU data structure are explored, i.e. the taxonomy, as well as, the correlation between OTUs to create similarity between adjacent OTUs. Spearman rank coefficient matrix of the OTUs within each phyla cluster is used to capture similarity in adjacent OTUs. Each row of the Spearman rank coefficient matrix(p × p matrix for *p* OTUs in a cluster) is reduced to a cumulative correlation coefficient, using the formula:


(2) $$\begin{array}{c} \rho_{OTU_{row_{j}}}=\sqrt[{p}]{\left|\rho_{OTU_{j1}}|\cdot |\rho_{OTU_{j2}}|\ldots .\cdot |\rho_{OTU_{jp}}\right|} \end{array}$$

for j∈[1,p].

**Figure 4.**
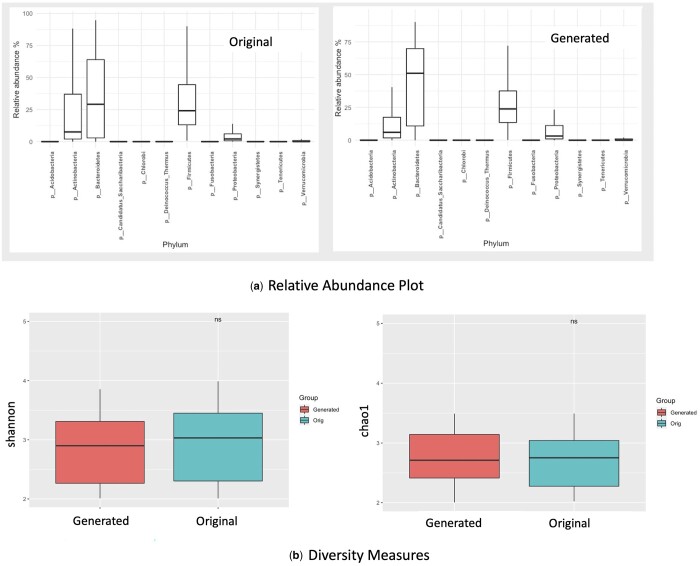
Diversity metrics and relative abundance plots comparing data generated through T2D dataset. (a) Relative abundance plot showing abundance at the phylum level is very closely modeled in the generated data as compared to the original data. (b) Box plots of Shannon index and Chao 1 index calculated from real data and generated datasets. The comparison of boxplots using Wilcox test showed *P* value >0.05 (showed as ns) indicating the diversity metrics for the generated and original data are similar.

Further, each of these cumulative coefficients are arranged in a decreasing order forming a sorted vector $P_{\mathrm{OTU}}^{*}$ using the formula:


(3) $$\begin{array}{c} P_{\mathrm{OTU}}^{*}=\{\rho_{OTU_{\mathrm{row}5}},\rho_{OTU_{\mathrm{row}3}}\ldots ..,\rho_{OTU_{row_{p}}}\} \end{array}$$

Subsequently, the heatmap obtained by the correlations in the OTU data is re-ordered based on the decreasing order of the cumulative correlation coefficients. Through this ordering the correlation structure between the OTUs is used to establish a similarity in the neighboring OTUs before being provided to the CNN model. This variation of CNN modeling is named as $\mathrm{taxoN}N_{\mathrm{corr}}$ (Sharma *et al.* 2020). The CNN is applied individually to each phyla group and features are extracted from the major phyla groups (refer Supplementary Fig. S9 for CNN’s architecture). Next, ensemble learning (Hansen and Salamon 1990) is used, where, features from each group are combined. The flattened feature vectors obtained from each group are merged via concatenation to make one very long vector that is further passed through a softmax function for final prediction of binary status of disease. The loss for our CNN is computed using weighted cross entropy loss to account for class imbalance if any between cases and controls. The functional working of the layers in the ensemble based CNN network is provided in Supplementary Fig. S9.

## 3 Results

### 3.1 Data description

**Figure 5.**
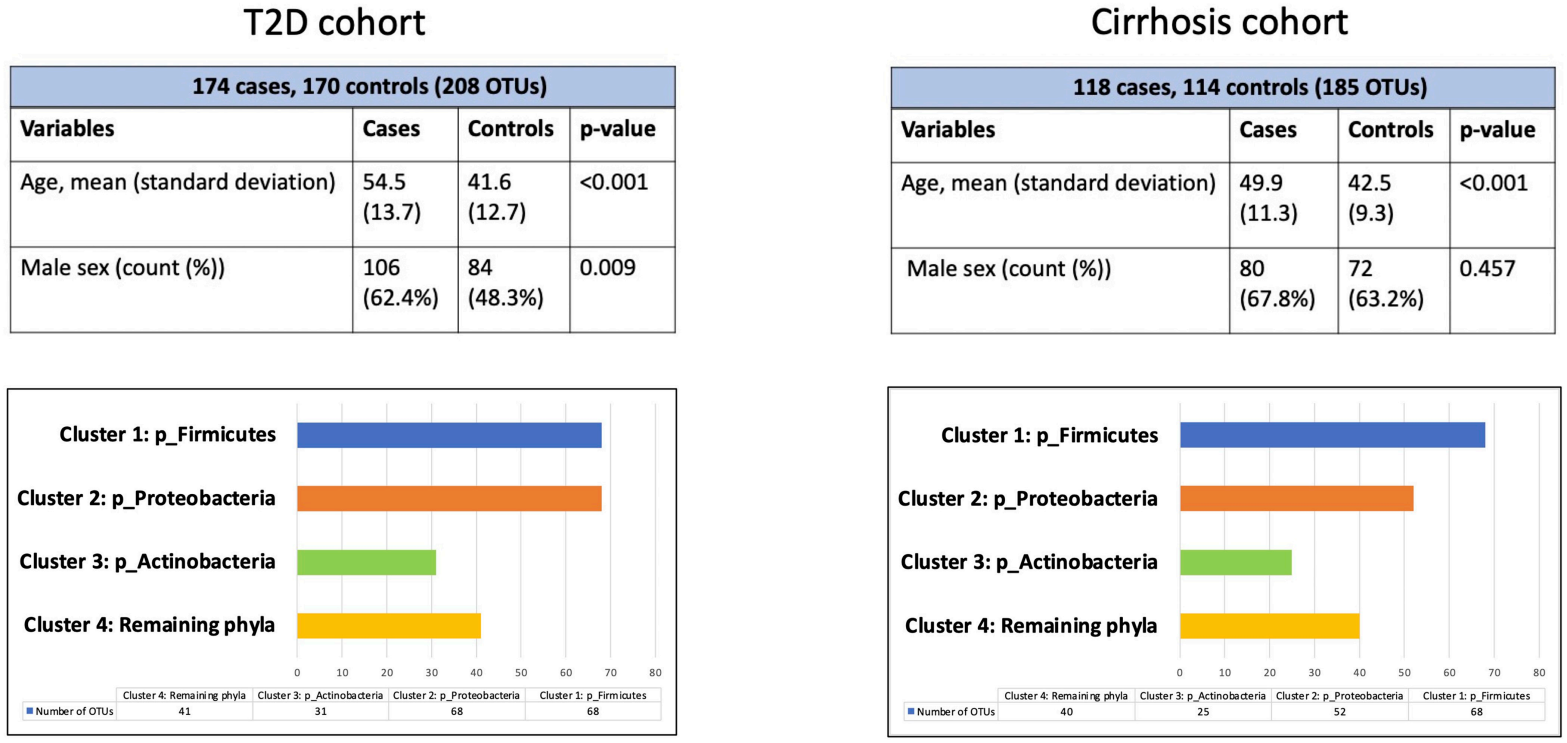
Cluster wise stratification of T2D and Cirrhosis datasets.

To assess the predictive ability of phylaGAN on linking the gut microbiome with disease risk, we implemented our algorithm on two main datasets T2D (Qin *et al.* 2012) study containing 174 cases and 170 controls and a Cirrhosis study (Qin *et al.* 2014), containing 118 cases and 114 controls (refer Fig. 5). In addition, we externally validated our model on a study by Le Chatelier *et al.* (2013) containing 164 obese cases and 89 lean controls. In all studies OTUs at the genus level in the kingdom ‘Bacteria’ were used as an input. The T2D data was based on deep next-generation shotgun sequencing of DNA extracted from the stool samples from Chinese subjects. The subjects in the Cirrhosis data were of Han Chinese origin and those of the study by Le Chatelier *et al.* were of Danish origin. In all three studies, following an analysis of accuracy in generating various sample sizes, as depicted in Supplementary Table S8, we generated 600 cases and 600 controls each using our phylaGAN model. Proteobacteria, Actinobacteria and Firmicutes emerged as the phyla with majority of OTUs, leading to forming three major clusters for $\mathrm{taxoN}N_{\mathrm{corr}}$ methodology. Supplementary Tables S2–S4 give more details about the OTUs in each cluster in the T2D, Cirrhosis and Le Chatelier *et al.* study respectively. The box-plots containing relative abundance percentages of OTUs in each phylum of T2D and Cirrhosis study are presented in Supplementary Figs S1 and S5 respectively. Supplementary Figs S2–S4 and Supplementary Figs S6–S8 provide boxplots for relative abundance percentages of genera in each cluster of the T2D and Cirrhosis study.

### 3.2 Experimental setting

For the neural network modeling, the datasets were randomly divided into 70% as the training data to train the *phylaGAN* model and 30% as the independent test data to evaluate the model performance. An internal validation was done using 10 times 10-fold cross validation on the training set, to analyze model performance before testing and to eliminate overfitting. For each fold of the cross-validation, 90% of the total training set was selected at random for training, and the remaining 10% was selected as a hold out set for validation. We obtained 10 Area Under the Curve (AUC) values corresponding to initial 10 folds in the training set. We repeated this process 10 times in order to generate corresponding 100 AUC values. We then calculated the 95% confidence intervals using these 100 AUC values. An external validation was also carried out on the Le Chatelier study classifying between obese and lean controls, where through transfer learning we use the GAN models trained on the T2D and Cirrhosis dataset to augment data on the external dataset. 400 epochs were run for the neural network model with a stride size of 1, batch size of 5 and number of filters in the CNN network as 32. Each network was trained using stochastic gradient descent with a learning rate of 0.001. We trained our network on an NVIDIA Tesla P100 GPU with 16 GB of RAM using tensorflow library in Python along with some data analysis using R version 3.5.3. The performance of our technique was evaluated through mean Area Under the Receiver Operating Characteristics Curve (AUC), the 95% Confidence Intervals of the AUC values, Sensitivity, Specificity, Positive Predictive Value (PPV) and Negative Predictive Value (NPV).

We compared the results using augmented dataset obtained through our proposed framework on ML models, $\mathrm{taxoN}N_{\mathrm{corr}}$ (Sharma *et al.* 2020), Random Forests (RF) (Liaw *et al.* 2002), Ridge regression (Hoerl and Kennard 1970), Lasso regression (Tibshirani 1996), Support Vector Machines (SVM) (Suykens and Vandewalle 1999), Gaussian Bayes Classifier (GBC) (Hand and Yu 2001) and Naive Bayes (NB) (Rish 2001). Hyperparameter tuning for each of the above mentioned models is provided in Supplementary Tables S2 and S3.

### 3.3 Evaluation of generated microbiome data properties

First, we evaluated sparsity in the generated samples, which is the proportion of zeros in a sample. For the real data, the observed sparsity in the controls was 0.75 [0.65, 0.89] and for the case group it was observed to be 0.79 [0.70, 0.91] respectively. As for the generated data in the T2D dataset, the sparsity was observed to be 0.77 [0.68, 0.87] and for Cirrhosis dataset was observed to be 0.76 [0.67, 0.85] which is comparable to the original datasets for both groups. Thus, *phylaGAN* simulated data could better capture the sparsity observed across all the samples of the real data. This conclusion also held for the smaller sample size case in the Le Chatelier *et al.* study where sparsity was observed to be 0.75 [0.71, 0.88].

Next, we evaluated the diversity in terms of shannon and chao index for the original and generated dataset. As seen in Fig. 4b we observed that the *P* value was >0.05 showing that both datasets are quite similar in terms of diversity of the microbiome data.

### 3.4 Evaluation of predictive performance

In this section we present results on the test sets of the augmented T2D, Cirrhosis and the external validation study by. We filtered the data in both studies, eliminating OTUs that had zeroes in all individuals and thereby obtained 184 OTUs for the Cirrhosis study and 208 for the T2D study after this filtering. Illustration of how the heatmaps are sorted and rearranged based on the correlations between the OTUs in each cluster are provided in Supplementary Figs S10–S12 for T2D and Supplementary Figs S13–S15 for Cirrhosis study.

#### 3.4.1 Results for T2D study

The results in terms of mean AUC and 95% C.I. for T2D dataset taking 10-fold cross validation on the test set are presented in column 2 of Supplementary Table S5. On the test set, the mean AUC values obtained for the augmented dataset for $\mathrm{taxoN}N_{\mathrm{corr}}$ approach were 0.812 as compared to 0.733 using the original dataset followed by Random Forest (AUC = 0.799), SVM (AUC = 0.742), Ridge regression (AUC = 0.722), Lasso regression (AUC = 0.695), GBC (AUC = 0.688) and NB (AUC = 0.701). These results as tabulated in Table 1 show that on performance metrics such as mean AUC, Sensitivity, Specificity, PPV and NPV showed that *phylaGAN* augmented data yielded considerably better performance metrics as compared to using the original dataset.

**Table 1.** Performance metric values [AUC, Sensitivity, Specificity, Positive Predictive value (PPV), Negative Predictive Value (NPV)] tabulated for various machine learning methods on test set of T2D study.

Methods	AUC		Sensitivity		Specificity		PPV		NPV	
	*Original data*	PhylaGAN augmented data	*Original data*	PhylaGAN augmented data	*Original data*	PhylaGAN augmented data	*Original data*	PhylaGAN augmented data	*Original data*	PhylaGAN augmented data
**taxoNN_corr**	***0.733***	**0.812**	***0.651***	**0.750**	***0.815***	**0.822**	***0.668***	**0.766**	***0.786***	**0.829**
RF	*0.703*	0.742	*0.621*	0.711	*0.785*	0.776	*0.638*	0.726	*0.756*	0.788
SVM	*0.701*	0.729	*0.619*	0.703	*0.783*	0.731	*0.636*	0.723	*0.754*	0.738
Lasso	*0.665*	0.695	*0.583*	0.677	*0.747*	0.699	*0.6*	0.711	*0.718*	0.701
Ridge	*0.700*	0.722	*0.618*	0.695	*0.782*	0.720	*0.635*	0.726	*0.753*	0.732
GBC	*0.642*	0.688	*0.56*	0.65	*0.724*	0.696	*0.577*	0.689	*0.695*	0.70
NB	*0.682*	0.701	*0.6*	0.680	*0.764*	0.713	*0.617*	0.684	*0.735*	0.723

The italicized columns represent the performance on original dataset. The non-italicized columns represent performance on the augmented dataset. The top row in bold shows the stratified CNN model ($\mathrm{taxoN}N_{\mathrm{corr}}$) performs better than all other ML approaches for disease prediction.

#### 3.4.2 Results for Cirrhosis study

The results for Cirrhosis study taking 10 times 10-fold cross validation by creating 10 folds in the training set and using 1 out of the 10 folds for testing each time are presented in column 3 of Supplementary Table S5. For the test set mean AUC values were calculated for the other machine learning methods in comparison to $\mathrm{taxoN}N_{\mathrm{corr}}$ for the augmented and original datasets. As shown in Table 2, we obtained a mean AUC value as high as 0.952 for the $\mathrm{taxoN}N_{\mathrm{corr}}$ model followed by the Random Forest (AUC = 0.913) and SVM method which was observed to give a mean AUC of 0.900. The Lasso and Ridge regression performed comparably with mean AUC values close to 0.865. The GBC and NB methods provided a mean AUC of 0.830 and 0.822 respectively. The observed AUC values showed the effectiveness of using augmented dataset on all the ML approaches for prediction on the Cirrhosis study.

**Table 2.** Performance metric values [AUC, Sensitivity, Specificity, Positive Predictive value (PPV), Negative Predictive Value (NPV)] tabulated for various machine learning methods on test set of Cirrhosis study.

Methods	AUC		Sensitivity		Specificity		PPV		NPV	
	*Original data*	PhylaGAN augmented data	*Original data*	PhylaGAN augmented data	*Original data*	PhylaGAN augmented data	*Original data*	PhylaGAN augmented data	*Original data*	PhylaGAN augmented data
**taxoNN_corr**	***0.911***	**0.952**	***0.846***	**0.899**	***0.890***	**0.943**	***0.858***	**0.883**	***0.924***	**0.939**
RF	*0.893*	0.913	*0.828*	0.851	*0.872*	0.926	*0.840*	0.826	*0.906*	0.918
SVM	*0.877*	0.900	*0.812*	0.843	*0.856*	0.891	*0.824*	0.823	*0.890*	0.908
Lasso	*0.823*	0.866	*0.758*	0.827	*0.802*	0.879	*0.770*	0.811	*0.836*	0.891
Ridge	*0.842*	0.865	*0.777*	0.812	*0.821*	0.880	*0.789*	0.826	*0.855*	0.882
GBC	*0.816*	0.830	*0.751*	0.821	*0.795*	0.876	*0.763*	0.829	*0.828*	0.878
NB	*0.802*	0.822	*0.737*	0.810	*0.781*	0.863	*0.749*	0.814	*0.815*	0.858

The italicized columns represent the performance on original dataset. The non-italicized columns represent performance on the augmented dataset. The top row in bold shows the stratified CNN model ($\mathrm{taxoN}N_{\mathrm{corr}}$) performs better than all other ML approaches for disease prediction.

#### 3.4.3 Results using transfer learning applied to study by Le Chatelier *et al.*

For the study by Le Chatelier *et al.*, the results taking 10 times 10-fold cross validation are presented in column 4 of Supplementary Table S5. In the test set performance we observed that on this small dataset, after augmentation, the performance improved dramatically from a mean AUC of 0.612–0.810 (improvement of 32%) for the $\mathrm{taxoN}N_{\mathrm{corr}}$ model, followed in performance by the Random Forest (AUC = 0.745), SVM method (AUC = 0.729). The Lasso and Ridge regression performed comparably with mean AUC values of 0.722 and 0.723 respectively. Results on the test set of the study by Chatelier *et al.* are reported in Table 3, showing the dramatic improvement observed by using transfer learning approach and the augmented dataset instead of the small original dataset in this study.

**Table 3.** Performance metric values [AUC, Sensitivity, Specificity, Positive Predictive value (PPV), Negative Predictive Value (NPV)] tabulated for various machine learning methods on test set of the study by Le Chatelier *et al.*

Methods	AUC		Sensitivity		Specificity		PPV		NPV	
	*Original data*	PhylaGAN augmented data	*Original data*	PhylaGAN augmented data	*Original data*	PhylaGAN augmented data	*Original data*	PhylaGAN augmented data	*Original data*	PhylaGAN augmented data
**taxoNN_corr**	***0.612***	**0.810**	***0.665***	**0.778**	***0.656***	**0.814**	***0.635***	**0.783**	***0.647***	**0.827**
RF	*0.600*	0.745	*0.653*	0.722	*0.644*	0.756	*0.623*	0.726	*0.635*	0.827
SVM	*0.597*	0.729	*0.650*	0.713	*0.641*	0.731	*0.620*	0.723	*0.632*	0.728
Lasso	*0.592*	0.722	*0.645*	0.717	*0.636*	0.729	*0.615*	0.711	*0.627*	0.730
Ridge	*0.598*	0.723	*0.651*	0.711	*0.642*	0.720	*0.621*	0.726	*0.633*	0.722
GBC	*0.575*	0.678	*0.628*	0.680	*0.619*	0.686	*0.598*	0.709	*0.610*	0.706
NB	*0.523*	0.675	*0.576*	0.653	*0.567*	0.683	*0.546*	0.704	*0.558*	0.708

The italicized columns represent the performance on original dataset. The non-italicized columns represent performance on the augmented dataset. The top row in bold shows the stratified CNN model ($\mathrm{taxoN}N_{\mathrm{corr}}$) performs better than all other ML approaches for disease prediction.

## 4 Discussion

While microbiome analysis greatly facilitates the investigation of association between the human microbiome and diseases, the evaluation of existing models using Machine Learning requires large amount of data. Similarly, it is challenging to specify explicit statistical distributions in simulation to fully mimic the complex patterns observed in real microbiome data. To address these challenges, we have developed a novel augmentation framework, *phylaGAN*. It adapts phylogenetic transformation to simulate microbiome relative abundances that are not easily distinguishable from real data in terms of sample-level characteristics and taxa–taxa relationships. The novelty in our approach toward using autoencoder to create more similarity between the real and generated data also makes our data appropriate for prediction tasks. We also show promising results in transfer learning on a small dataset where, we use our trained model to generate samples on datasets with limited sample size justifying our method’s generalizability on varied datasets.

We have provided extensive analyses of the performance of several machine learning methods on the two test datasets and an external validation set as discussed in Section 3.2. We show that the ML approaches perform considerably better with the augmented dataset. We also analyzed the methods in the literature that propose machine learning techniques for disease prediction for T2D and Cirrhosis studies. In study by Qin *et al.* (2014), an SVM method is used with training set (AUC of 0.918) and leave-one-out cross-validation set (AUC of 0.838) for the Cirrhosis data. In comparison, on our augmented dataset’s test set our method outperformed by a significant margin giving an AUC value of 0.952. In study by Qin *et al.* (2012), to exploit the potential ability of T2D classification by gut microbiota, a T2D classifier system based on the 50 gene markers through a minimum redundancy–maximum relevance (mRMR) feature selection method is proposed. An AUC of 0.81 was reported by using SVM for classification through the gene markers. As our model focused on relative abundance of the OTUs, therefore, a direct comparison to the results provided in study by Qin *et al.* (2012) was not feasible. Through Integrated Gradient methodology (Sundararajan *et al.* 2017) we are also able to provide the top-10 important OTUs as obtained through the neural network modeling for predicting disease status in both T2D and Cirrhosis studies, paving way for explainable AI (refer Supplementary Fig. S16).

Apart from the strengths, there are a few limitations of our method. Microbiomes can reside in various sites in the body such as skin, mammary glands, uterus, ovarian follicles, oral mucosa and gut. However, for the scope of this paper, we implemented our algorithm only on gut microbiome data. Thus, we have limited our analysis to predicting diseases caused by gut microbiomes. Another limitation is that although we show that through transfer learning on a smaller dataset our approach performs well however, it is under the assumption that the pipeline for sequencing the microbiome data needs to be the same on the smaller dataset such that the transfer learning can be appropriately applied. Also, in our methodology, we have generated balanced number of cases and controls (600 subjects respectively) to make the neural network learning unbiased, however, in the future, to model real life microbiome datasets, imbalanced case controls can be generated as well and further weighted neural network learning can be used to tackle the imbalance (refer Supplementary Table S8). In addition, although usage of autoencoder paves way for improvement of prediction performance, in the future, more methodological advancement toward making the generated samples closer to the original dataset and increasing the generative performance with low sample size should be carried out.

Our proposed framework, *phylaGAN* can benefit future microbiome research where researchers can use *phylaGAN* to simulate microbiome abundances for a certain sample size preserving important OTU relationships, as well as, through autoencoder approach can bridge the gap between original and simulated data to have an effective representation of augmented microbiome data for prediction tasks. In conclusion, *phylaGAN* framework enables the evaluation of various types of microbiome studies by providing simulated data with high fidelity to the real data. In addition, machine learning models that have large dataset requirements for proper training can benefit from the use *phylaGAN* framework for model evaluation and prediction performance improvement.

## Supplementary Material

btae161_Supplementary_Data
