# Supplementary material for: phylaGAN: data augmentation through conditional GANs and autoencoders for improving disease prediction accuracy using microbiome data
Source: Bioinformatics. 2024 Apr 3;40(4):btae161. doi: 10.1093/bioinformatics/btae161 (PMC11256914; doi:10.1093/bioinformatics/btae161)
Supplement: btae161_Supplementary_Data [file btae161_supplementary_data.pdf]

Supplementary Material for 'phylaGAN: Data augmentation through Conditional GANs and Autoencoders for improving disease prediction accuracy using microbiome data'

Contents

1 Supplementary Tables 5

2 Supplementary Figures 28

3 Supplementary Methods 44

3.1 Extracting important features through Integrated Gradient Methodology 44

4 References 44

List of Supplementary Tables

1 Parameter Values for Encoder Network 6

2 Hyperparameters and their search range and tuned values for CNN modeling (taxoNNcorr) 7

3 Hyperparameters and their search range and tuned values for the conventional Machine Learning models 8

4 Table detailing the clusters in the T2D study [1] based on the phyla containing maximum number of OTUs. The right handside represents the genera in each cluster. The numbering provided to each genus provides a unique identifier to each OTU which is further used in Heatmaps as labels for the x and y axis, in Supplementary Figure 10, 11 and 12 to illustrate the correlations between the OTUs. 9

5 Table detailing the clusters in the Cirrhosis study [2] based on the phyla containing maximum number of OTUs. The right handside represents the genera in each cluster. The numbering provided to each genus provides a unique identifier to each OTU which is further used in Heatmaps as labels for the x and y axis, in Supplementary Figure 13, 16 and 15 to illustrate the correlations between the OTUs. 15

6 Table detailing the clusters in the LeChatelier et al. study [3] based on the phyla containing maximum number of OTUs. The right handside represents the genera in each cluster. The numbering provided to each genus provides a unique identifier to each OTU which is further used in Heatmaps as labels for the x and y axis, in Supplementary Figure 10, 11 and 12 to illustrate the correlations between the OTUs. 20

7 95% confidence intervals obtained for the mean AUC values for 10 times 10-fold cross validation on the training set for the (a) T2D study (b) Cirrhosis study, (c) Le Chatelier study (Obesity study) 26

8 AUC values obtained for both T2D and Cirrhosis studies with varying numbers of generated cases and controls. The last three rows illustrate imbalanced ratios of cases to controls (ranging from 1:3 to 3:4), demonstrating the method's robustness in handling class imbalance. The best AUC is highlighted in bold. 27

List of Supplementary Figures

1 Boxplot illustrating relative abundance percentage of OTUs in each phylum of the T2D study. The upper whisker extends from the hinge to the largest value no further than 1.5 * IQR from the hinge (where IQR is the inter-quartile range, or distance between the first and third quartiles). The lower whisker extends from the hinge to the smallest value at most 1.5 * IQR of the hinge. Data beyond the end of the whiskers are called "outlying" points and are plotted individually. 29

2 Relative abundance percentage of OTUs at genus level in the Firmicutes phylum of the T2D study 30

3 Relative abundance percentage of OTUs at genus level in the Proteobacteria phylum of the T2D study 31

4 Relative abundance percentage of OTUs at genus level in the Actinobacteria phylum of the T2D study 32

5 Boxplot illustrating relative abundance percentage of OTUs in each phylum of the Cirrhosis study. The upper whisker extends from the hinge to the largest value no further than 1.5 * IQR from the hinge (where IQR is the inter-quartile range, or distance between the first and third quartiles). The lower whisker extends from the hinge to the smallest value at most 1.5 * IQR of the hinge. Data beyond the end of the whiskers are called ”outlying” points and are plotted individually. 33

6 Relative abundance percentage of OTUs at genus level in the Firmicutes phylum of the Cirrhosis study 34

7 Relative abundance percentage of OTUs at genus level in the Proteobacteria phylum of the Cirrhosis study 35

8 Relative abundance percentage of OTUs at genus level in the Actinobacteria phylum of the Cirrhosis study 36

9 Functional working of the layers of $taxoNN$ on 4 clusters of an example dataset containing ‘p’, ‘q’, ‘r’ and ‘s’ OTUs in the respective clusters (where p+q+r+s = N). Each block corresponds to a layer acting on the cluster. Input signifies the dimension of the input to the layer. The input at each step is represented as (k,l) where, ‘k’ is the number of rows in the input and ‘l’ represents the number of columns. As the initial input was a vector therefore, l in this case was ‘1’. Output signifies the dimension of the result after certain operations in that particular layer. Further, as the number of filters increases from 32 in the first Conv layer to 64 in the second Conv layer, the number of columns in the nodes vary from 32 to 64. Finally, in the concatenation step we obtain a single column concatenation vector by stacking flattened vectors from all clusters together. 37

10 Heatmaps for the Spearman rank of the OTUs in the cluster, Phylum Firmicutes, (a) before ordering and (b) after the ordering based on correlation of the OTUs in the T2D study 38

11 Heatmaps for the Spearman rank of the OTUs in the cluster, Phylum Proteobacteria, (a) before ordering and (b) after the ordering based on correlation of the OTUs in the T2D study 39

12 Heatmaps for the Spearman rank of the OTUs in the cluster, Phylum Actinobacteria, (a) before ordering and (b) after the ordering based on correlation of the OTUs in the T2D study 40

13 Heatmaps for the Spearman rank of the OTUs in the cluster, Phylum Firmicutes, (a) before ordering and (b) after the ordering based on correlation correlation of the OTUs in the Cirrhosis study 41

14 Heatmaps for the Spearman rank of the OTUs in the cluster, Phylum Proteobacteria, (a) before ordering and (b) after the ordering based on correlation correlation of the OTUs in the Cirrhosis study 42

15 Heatmaps for the Spearman rank of the OTUs in the cluster, Phylum Actinobacteria, (a) before ordering and (b) after the ordering based on correlation correlation of the OTUs in the Cirrhosis study 43

16 Identifying variable importance through IG approach in NN modeling. (a) Top-10 important OTUs at the genus level for predicting disease status in the Cirrhosis study and (b) Top-10 important OTUs at the genus level for predicting disease status in the T2D study. The higher the gradient more important the feature. 44

1 Supplementary Tables

Supplementary Table 1: Parameter Values for Encoder Network

**Component**	**Parameter**	**Value**
Encoder	No. filters in the 1st layer	256
	No. filters in the 2nd layer	256
	No. filters in the 3rd layer	128
	No. filters in the 4th layer	64
Decoder	No. filters in the 1st layer	64
	No. filters in the 2nd layer	128
	No. filters in the 3rd layer	256
	No. filters in the 4th layer	256

Supplementary Table 2: Hyperparameters and their search range and tuned values for CNN modeling (taxoNNcorr)

**Hyperparameter**	**Value**	**Search Range**
Kernel size for Convolution	20	3, 7, 15, 20, 30, 40, 50
Pooling method	Max pooling	Max pooling, Average pooling
Number of units in hidden layer	400	100-600
Feature Scaling	Standard	Min-Max and Standard scaler
Number of Layers	3	1,3,5,7
Hidden units/ layer<br>Number of epochs	32<br>400	12,24,32,48,64,96,192<br>100-600
Dropout rate	0.15	[0,0.8]
Learning Rate	0.005	[0.0001,0.1]
Optimization solver	Adam	Stochastic Gradient Descent, Adam

Supplementary Table 3: Hyperparameters and their search range and tuned values for the conventional Machine Learning models

Approaches	Parameter	Value	Search Range
Random Forest	No. of Trees	400	[10, 500]
	Mtry (No. of columns to randomly select at each level)	380	[50,400]
SVM Classifier	C	1	[0.1, 100]
	Kernel	sigmoid	'linear', 'poly', 'rbf', 'sigmoi[illegible]
Lasso Regression	alpha	0.005	[0.001, 0.1]
Ridge Regression	alpha	0.005	[0.001, 0.1]

Supplementary Table 4: Table detailing the clusters in the T2D study [1] based on the phyla containing maximum number of OTUs. The right handside represents the genera in each cluster. The numbering provided to each genus provides a unique identifier to each OTU which is further used in Heatmaps as labels for the x and y axis, in Supplementary Figure 10, 11 and 12 to illustrate the correlations between the OTUs.

**OTUs in T2D study**		
**Cluster**	**Phylum**	**Genus**
Cluster 1	p_Firmicutes	1. g_Abiotrophia<br>2. g_Acidaminococcaceae_unclassified<br>3. g_Acidaminococcus<br>4. g_Alicyclobacillus<br>5. g_Allobaculum<br>6. g_Anaerococcus<br>7. g_Anaerofustis<br>8. g_Anaeroglobus<br>9. g_Anaerostipes<br>10. g_Anaerotruncus<br>11. g_Bacillus<br>12. g_Blautia<br>13. g_Bulleidia<br>14. g_Butyricicoccus<br>15. g_Butyrivibrio<br>16. g_Catenibacterium<br>17. g_Cellulosilyticum<br>18. g_Clostridiaceae_noname<br>19. g_Clostridiales_Family_XIII_Incertae_Sedis_noname<br>20. g_Clostridiales_Family_XIII_Incertae_Sedis_unclassified<br>21. g_Clostridiales_noname<br>22. g_Clostridium<br>23. g_Coprobacillus<br>24. g_Coprococcus<br>25. g_Dialister<br>26. g_Dorea<br>27. g_Eggerthia<br>28. g_Enterococcus<br>29. g_Erysipelotrichaceae_noname<br>30. g_Eubacterium<br>31. g_Faecalibacterium<br>32. g_Finegoldia

		33. g_Flavonifractor<br>34. g_Gemella<br>35. g_Granulicatella<br>36. g_Holdemania<br>37. g_Lachnoanaerobaculum<br>38. g_Lachnospiraceae_noname<br>39. g_Lactobacillus<br>40. g_Lactococcus<br>41. g_Leuconostoc<br>42. g_Marvinbryantia<br>43. g_Megamonas<br>44. g_Megasphaera<br>45. g_Mitsuokella<br>46. g_Oribacterium<br>47. g_Oscillibacter<br>48. g_Parvimonas<br>49. g_Pediococcus<br>50. g_Peptoniphilus<br>51. g_Peptostreptococcaceae_noname<br>52. g_Peptostreptococcus<br>53. g_Phascolarctobacterium<br>54. g_Pseudoflavonifractor<br>55. g_Pseudoramibacter<br>56. g_Roseburia<br>57. g_Ruminococcaceae_noname<br>58. g_Ruminococcus<br>59. g_Selenomonas<br>60. g_Shuttleworthia<br>61. g_Solobacterium<br>62. g_Staphylococcus<br>63. g_Stomatobaculum<br>64. g_Streptococcus<br>65. g_Subdoligranulum<br>66. g_Turicibacter<br>67. g_Veillonella<br>68. g_Weissella
Cluster 2	p_Proteobacteria	69. g_Acinetobacter<br>70. g_Actinobacillus<br>71. g_Aeromonas<br>72. g_Aggregatibacter<br>73. g_Bartonella<br>74. g_Bilophila

		75. g_Brevundimonas
		76. g_Buchnera
		77. g_Burkholderia
		78. g_Burkholderiales_noname
		79. g_Campylobacter
		80. g_Candidatus_Zinderia
		81. g_Cardiobacteriaceae_unclassified
		82. g_Caulobacter
		83. g_Chromobacterium
		84. g_Citrobacter
		85. g_Citromicrobium
		86. g_Comamonas
		87. g_Cronobacter
		88. g_Cupriavidus
		89. g_Desulfovibrio
		90. g_Enhydrobacter
		91. g_Enterobacter
		92. g_Enterobacteriaceae_noname
		93. g_Erythrobacteraceae_unclassified
		94. g_Escherichia
		95. g_Gallionellaceae_unclassified
		96. g_Haemophilus
		97. g_Halomonas
		98. g_Helicobacter
		99. g_Kingella
		100. g_Klebsiella
		101. g_Lautropia
		102. g_Limnohabitans
		103. g_Mesorhizobium
		104. g_Morganella
		105. g_Neisseria
		106. g_Oxalobacter
		107. g_Pantoea
		108. g_Paracoccus
		109. g_Parasutterella
		110. g_Plesiomonas
		111. g_Polaromonas
		112. g_Proteus
		113. g_Providencia
		114. g_Pseudoalteromonadaceae_unclassified
		115. g_Pseudoalteromonas
		116. g_Pseudomonas

		117. g_Pseudoxanthomonas<br>118. g_Raoultella<br>119. g_Rheinheimera<br>120. g_Rhodanobacter<br>121. g_Rhodobiaceae_unclassified<br>122. g_Serratia<br>123. g_Shewanella<br>124. g_Shigella<br>125. g_Shinella<br>126. g_Sinobacteraceae_unclassified<br>127. g_Sphingobium<br>128. g_Sphingopyxis<br>129. g_Spiribacter<br>130. g_Succinatimonas<br>131. g_Sutterella<br>132. g_Sutterellaceae_unclassified<br>133. g_Variovorax<br>134. g_Vibrio<br>135. g_Xanthomonas<br>136. g_Yersinia
Cluster 3	p_Actinobacteria	137. g_Actinomyces<br>138. g_Adlercreutzia<br>139. g_Agromyces<br>140. g_Alloscardovia<br>141. g_Atopobium<br>142. g_Bifidobacterium<br>143. g_Brachybacterium<br>144. g_Brevibacterium<br>145. g_Collinsella<br>146. g_Coriobacteriaceae_noname<br>147. g_Corynebacterium<br>148. g_Cryptobacterium<br>149. g_Dermatophilaceae_unclassified<br>150. g_Eggerthella<br>151. g_Gardnerella<br>152. g_Gordonibacter<br>153. g_Kocuria<br>154. g_Leifsonia<br>155. g_Leucobacter<br>156. g_Microlunatus<br>157. g_Mobiluncus<br>158. g_Nocardioides

		159. g_Olsenella<br>160. g_Parascardovia<br>161. g_Propionibacteriaceae_unclassified<br>162. g_Propionibacterium<br>163. g_Rothia<br>164. g_Scardovia<br>165. g_Slackia<br>166. g_Tropheryma<br>167. g_Varibaculum
Cluster 4	1. p_Spirochaetes<br>2. p_Synergistetes<br>3. p_Tenericutes<br>4. p_Verrucomicrobia<br>5. p_Bacteroidetes<br>6. p_Candidatus _Saccharibacteria<br>7. p_Chlorobi<br>8. p_Deinococcus _Thermus<br>9. p_Acidobacteria<br>10. p_Fusobacteria	168. g_Brachyspira<br>169. g_Fretibacterium<br>170. g_Pyramidobacter<br>171. g_Synergistes<br>172. g_Mycoplasma<br>173. g_Akkermansia<br>174. g_Naumovozyma<br>175. g_Saccharomyces<br>176. g_Saccharomycetaceae_unclassified<br>177. g_Alistipes<br>178. g_Alloprevotella<br>179. g_Bacteroidales_noname<br>180. g_Bacteroides<br>181. g_Bacteroidetes_noname<br>182. g_Barnesiella<br>183. g_Butyricimonas<br>184. g_Cellulophaga<br>185. g_Coprobacter<br>186. g_Dysgonomonas<br>187. g_Odoribacter<br>188. g_Parabacteroides<br>189. g_Paraprevotella<br>190. g_Pedobacter<br>191. g_Porphyromonas<br>192. g_Prevotella<br>193. g_Riemerella<br>194. g_Sphingobacterium<br>195. g_Zunongwangia<br>196. g_Candidatus_Saccharibacteria_noname<br>197. g_Candidatus_Saccharibacteria_noname_unclassified

		198. g_Chlorobium<br>199. g_Deinococcus<br>200. g_Meiothermus<br>201. g_Methanocaldococcaceae_unclassified<br>202. g_Acidobacteriaceae_unclassified<br>203. g_Granulicella<br>204. g_Cetobacterium<br>205. g_Fusobacterium<br>206. g_Leptotrichia<br>207. g_Leptotrichiaceae_unclassified<br>208. g_Rhodopirellula

Supplementary Table 5: Table detailing the clusters in the Cirrhosis study [2] based on the phyla containing maximum number of OTUs. The right handside represents the genera in each cluster. The numbering provided to each genus provides a unique identifier to each OTU which is further used in Heatmaps as labels for the x and y axis, in Supplementary Figure 13, 16 and 15 to illustrate the correlations between the OTUs.

OTUs in Cirrhosis study		
**Cluster**	**Phylum**	**Genus**
Cluster 1	p_Firmicutes	1. g_Abiotrophia<br>2. g_Acidaminococcaceae_unclassified<br>3. g_Acidaminococcus<br>4. g_Aerococcus<br>5. g_Anaerococcus<br>6. g_Anaerofustis<br>7. g_Anaeroglobus<br>8. g_Anaerostipes<br>9. g_Anaerotruncus<br>10. g_Anoxybacillus<br>11. g_Bacillus<br>12. g_Blautia<br>13. g_Bulleidia<br>14. g_Butyricicoccus<br>15. g_Butyrivibrio<br>16. g_Catenibacterium<br>17. g_Catonella<br>18. g_Centipeda<br>19. g_Clostridiaceae_noname<br>20. g_Clostridiales_Family_XIII_Incertae_Sedis_noname<br>21. g_Clostridiales_Family_XIII_Incertae_Sedis_unclassified<br>22. g_Clostridiales_noname<br>23. g_Clostridium<br>24. g_Coprobacillus<br>25. g_Coprococcus<br>26. g_Dialister<br>27. g_Dorea<br>28. g_Eggerthia<br>29. g_Enterococcus<br>30. g_Erysipelotrichaceae_noname<br>31. g_Eubacterium<br>32. g_Faecalibacterium

		33. g_Filifactor
		34. g_Finegoldia
		35. g_Flavonifractor
		36. g_Gemella
		37. g_Granulicatella
		38. g_Holdemania
		39. g_Lachnoanaerobaculum
		40. g_Lachnospiraceae_noname
		41. g_Lactobacillus
		42. g_Lactococcus
		43. g_Leuconostoc
		44. g_Megamonas
		45. g_Megasphaera
		46. g_Mitsuokella
		47. g_Oribacterium
		48. g_Oscillibacter
		49. g_Parvimonas
		50. g_Pediococcus
		51. g_Peptoniphilus
		52. g_Peptostreptococcaceae_noname
		53. g_Peptostreptococcus
		54. g_Phascolarctobacterium
		55. g_Pseudoflavonifractor
		56. g_Roseburia
		57. g_Ruminococcaceae_noname
		58. g_Ruminococcus
		59. g_Selenomonas
		60. g_Shuttleworthia
		61. g_Solobacterium
		62. g_Staphylococcus
		63. g_Stomatobaculum
		64. g_Streptococcus
		65. g_Subdoligranulum
		66. g_Turicibacter
		67. g_Veillonella
		68. g_Weissella
Cluster 2	p_Proteobacteria	69. g_Acinetobacter
		70. g_Actinobacillus
		71. g_Aeromonas
		72. g_Aggregatibacter
		73. g_Bartonella
		74. g_Bilophila

		75. g_Bordetella<br>76. g_Burkholderia<br>77. g_Burkholderiales_noname<br>78. g_Campylobacter<br>79. g_Cardiobacteriaceae_unclassified<br>80. g_Cardiobacterium<br>81. g_Chromobacterium<br>82. g_Citrobacter<br>83. g_Comamonas<br>84. g_Cronobacter<br>85. g_Desulfovibrio<br>86. g_Eikenella<br>87. g_Enterobacter<br>88. g_Enterobacteriaceae_noname<br>89. g_Escherichia<br>90. g_Gallionellaceae_unclassified<br>91. g_Haemophilus<br>92. g_Halomonas<br>93. g_Helicobacter<br>94. g_Kingella<br>95. g_Klebsiella<br>96. g_Kosakonia<br>97. g_Lautropia<br>98. g_Morganella<br>99. g_Neisseria<br>100. g_Oxalobacter<br>101. g_Pantoea<br>102. g_Parasutterella<br>103. g_Pectobacterium<br>104. g_Plesiomonas<br>105. g_Proteus<br>106. g_Providencia<br>107. g_Pseudomonas<br>108. g_Pusillimonas<br>109. g_Ralstonia<br>110. g_Raoultella<br>111. g_Rhodopseudomonas<br>112. g_Rhodospirillum<br>113. g_Serratia<br>114. g_Shewanella<br>115. g_Shigella<br>116. g_Sinobacteraceae_unclassified	

		117. g_Succinatimonas
		118. g_Sutterella
		119. g_Sutterellaceae_unclassified
		120. g_Yersinia
Cluster 3	p_Actinobacteria	121. g_Actinomyces
		122. g_Actinopolyspora
		123. g_Adlercreutzia
		124. g_Alloscardovia
		125. g_Atopobium
		126. g_Bifidobacterium
		127. g_Brevibacterium
		128. g_Collinsella
		129. g_Coriobacteriaceae_noname
		130. g_Corynebacterium
		131. g_Cryptobacterium
		132. g_Eggerthella
		133. g_Gardnerella
		134. g_Gordonibacter
		135. g_Kocuria
		136. g_Olsenella
		137. g_Parascardovia
		138. g_Propionibacteriaceae_unclassified
		139. g_Propionibacterium
		140. g_Pseudonocardia
		141. g_Rothia
		142. g_Saccharomonospora
		143. g_Saccharopolyspora
		144. g_Scardovia
		145. g_Slackia
Cluster 4	1. p_Spirochaetes	146. g_Brachyspira
	2. p_Synergistetes	147. g_Fretibacterium
	3. p_Tenericutes	148. g_Pyramidobacter
	4. p_Verrucomicrobia	149. g_Synergistes
	5. p_Bacteroidetes	150. g_Akkermansia
	6. p_Candidatus _Saccharibacteria	151. g_Naumovozyma
	7. p_Chlorobi	152. g_Saccharomyces
	8. p_Deinococcus _Thermus	153. g_Saccharomycetaceae_unclassified
	9. p_Acidobacteria	154. g_Alistipes
	10. p_Fusobacteria	155. g_Alloprevotella

		156. g_Bacteroidales_noname
		157. g_Bacteroides
		158. g_Bacteroidetes_noname
		159. g_Barnesiella
		160. g_Butyricimonas
		161. g_Cellulophaga
		162. g_Coprobacter
		163. g_Dysgonomonas
		164. g_Odoribacter
		165. g_Parabacteroides
		166. g_Paraprevotella
		167. g_Pedobacter
		168. g_Porphyromonas
		169. g_Prevotella
		170. g_Riemerella
		171. g_Sphingobacterium
		172. g_Zunongwangia
		173. g_Candidatus_Saccharibacteria_noname
		174. g_Candidatus_Saccharibacteria_noname_unclassified
		175. g_Chlorobium
		176. g_Deinococcus
		177. g_Meiothermus
		178. g_Methanocaldococcaceae_unclassified
		179. g_Acidobacteriaceae_unclassified
		180. g_Granulicella
		181. g_Cetobacterium
		182. g_Fusobacterium
		183. g_Leptotrichia
		184. g_Leptotrichiaceae_unclassified

Supplementary Table 6: Table detailing the clusters in the LeChatelier et al. study [3] based on the phyla containing maximum number of OTUs. The right handside represents the genera in each cluster. The numbering provided to each genus provides a unique identifier to each OTU which is further used in Heatmaps as labels for the x and y axis, in Supplementary Figure 10, 11 and 12 to illustrate the correlations between the OTUs.

**OTUs in LeChatelier et al. study**		
**Cluster**	**Phylum**	**Genus**
Cluster 1	p_Firmicutes	1. g_Abiotrophia<br>2. g_Acidaminococcaceae_unclassified<br>3. g_Acidaminococcus<br>4. g_Alicyclobacillus<br>5. g_Allobaculum<br>6. g_Anaerococcus<br>7. g_Anaerofustis<br>8. g_Anaeroglobus<br>9. g_Anaerostipes<br>10. g_Anaerotruncus<br>11. g_Bacillus<br>12. g_Blautia<br>13. g_Bulleidia<br>14. g_Butyricicoccus<br>15. g_Butyrivibrio<br>16. g_Catenibacterium<br>17. g_Cellulosilyticum<br>18. g_Clostridiaceae_noname<br>19. g_Clostridiales_Family_XIII_Incertae_Sedis_noname<br>20. g_Clostridiales_Family_XIII_Incertae_Sedis_unclassified<br>21. g_Clostridiales_noname<br>22. g_Clostridium<br>23. g_Coprobacillus<br>24. g_Coprococcus<br>25. g_Dialister<br>26. g_Dorea<br>27. g_Eggerthia<br>28. g_Enterococcus<br>29. g_Erysipelotrichaceae_noname<br>30. g_Eubacterium<br>31. g_Faecalibacterium<br>32. g_Finegoldia

		33. g_Flavonifractor<br>34. g_Gemella<br>35. g_Granulicatella<br>36. g_Holdemania<br>37. g_Lachnoanaerobaculum<br>38. g_Lachnospiraceae_noname<br>39. g_Lactobacillus<br>40. g_Lactococcus<br>41. g_Leuconostoc<br>42. g_Marvinbryantia<br>43. g_Megamonas<br>44. g_Megasphaera<br>45. g_Mitsuokella<br>46. g_Oribacterium<br>47. g_Oscillibacter<br>48. g_Parvimonas<br>49. g_Pediococcus<br>50. g_Peptoniphilus<br>51. g_Peptostreptococcaceae_noname<br>52. g_Peptostreptococcus<br>53. g_Phascolarctobacterium<br>54. g_Pseudoflavonifractor<br>55. g_Pseudoramibacter<br>56. g_Roseburia<br>57. g_Ruminococcaceae_noname<br>58. g_Ruminococcus<br>59. g_Selenomonas<br>60. g_Shuttleworthia<br>61. g_Solobacterium<br>62. g_Staphylococcus<br>63. g_Stomatobaculum<br>64. g_Streptococcus<br>65. g_Subdoligranulum<br>66. g_Turicibacter<br>67. g_Veillonella<br>68. g_Weissella
Cluster 2	p_Proteobacteria	69. g_Acinetobacter<br>70. g_Actinobacillus<br>71. g_Aeromonas<br>72. g_Aggregatibacter<br>73. g_Bartonella<br>74. g_Bilophila

		75. g_Brevundimonas<br>76. g_Buchnera<br>77. g_Burkholderia<br>78. g_Burkholderiales_noname<br>79. g_Campylobacter<br>80. g_Candidatus_Zinderia<br>81. g_Cardiobacteriaceae_unclassified<br>82. g_Caulobacter<br>83. g_Chromobacterium<br>84. g_Citrobacter<br>85. g_Citromicrobium<br>86. g_Comamonas<br>87. g_Cronobacter<br>88. g_Cupriavidus<br>89. g_Desulfovibrio<br>90. g_Enhydrobacter<br>91. g_Enterobacter<br>92. g_Enterobacteriaceae_noname<br>93. g_Erythrobacteraceae_unclassified<br>94. g_Escherichia<br>95. g_Gallionellaceae_unclassified<br>96. g_Haemophilus<br>97. g_Halomonas<br>98. g_Helicobacter<br>99. g_Kingella<br>100. g_Klebsiella<br>101. g_Lautropia<br>102. g_Limnohabitans<br>103. g_Mesorhizobium<br>104. g_Morganella<br>105. g_Neisseria<br>106. g_Oxalobacter<br>107. g_Pantoea<br>108. g_Paracoccus<br>109. g_Parasutterella<br>110. g_Plesiomonas<br>111. g_Polaromonas<br>112. g_Proteus<br>113. g_Providencia<br>114. g_Pseudoalteromonadaceae_unclassified<br>115. g_Pseudoalteromonas<br>116. g_Pseudomonas	

		117. g_Pseudoxanthomonas<br>118. g_Raoultella<br>119. g_Rheinheimera<br>120. g_Rhodanobacter<br>121. g_Rhodobiaceae_unclassified<br>122. g_Serratia<br>123. g_Shewanella<br>124. g_Shigella<br>125. g_Shinella<br>126. g_Sinobacteraceae_unclassified<br>127. g_Sphingobium<br>128. g_Sphingopyxis<br>129. g_Spiribacter<br>130. g_Succinatimonas<br>131. g_Sutterella<br>132. g_Sutterellaceae_unclassified<br>133. g_Variovorax<br>134. g_Vibrio<br>135. g_Xanthomonas<br>136. g_Yersinia
Cluster 3	p_Actinobacteria	137. g_Actinomyces<br>138. g_Adlercreutzia<br>139. g_Agromyces<br>140. g_Alloscardovia<br>141. g_Atopobium<br>142. g_Bifidobacterium<br>143. g_Brachybacterium<br>144. g_Brevibacterium<br>145. g_Collinsella<br>146. g_Coriobacteriaceae_noname<br>147. g_Corynebacterium<br>148. g_Cryptobacterium<br>149. g_Dermatophilaceae_unclassified<br>150. g_Eggerthella<br>151. g_Gardnerella<br>152. g_Gordonibacter<br>153. g_Kocuria<br>154. g_Leifsonia<br>155. g_Leucobacter<br>156. g_Microlunatus<br>157. g_Mobiluncus<br>158. g_Nocardioides

		159. g_Olsenella<br>160. g_Parascardovia<br>161. g_Propionibacteriaceae_unclassified<br>162. g_Propionibacterium<br>163. g_Rothia<br>164. g_Scardovia<br>165. g_Slackia<br>166. g_Tropheryma<br>167. g_Varibaculum
Cluster 4	1. p_Spirochaetes<br>2. p_Synergistetes<br>3. p_Tenericutes<br>4. p_Verrucomicrobia<br>5. p_Bacteroidetes<br>6. p_Candidatus _Saccharibacteria<br>7. p_Chlorobi<br>8. p_Deinococcus _Thermus<br>9. p_Acidobacteria<br>10. p_Fusobacteria	168. g_Brachyspira<br>169. g_Fretibacterium<br>170. g_Pyramidobacter<br>171. g_Synergistes<br><br>172. g_Mycoplasma<br>173. g_Akkermansia<br><br>174. g_Naumovozyma<br>175. g_Saccharomyces<br><br>176. g_Saccharomycetaceae_unclassified<br>177. g_Alistipes<br>178. g_Alloprevotella<br>179. g_Bacteroidales_noname<br>180. g_Bacteroides<br>181. g_Bacteroidetes_noname<br>182. g_Barnesiella<br>183. g_Butyricimonas<br>184. g_Cellulophaga<br>185. g_Coprobacter<br>186. g_Dysgonomonas<br>187. g_Odoribacter<br>188. g_Parabacteroides<br>189. g_Paraprevotella<br>190. g_Pedobacter<br>191. g_Porphyromonas<br>192. g_Prevotella<br>193. g_Riemerella<br>194. g_Sphingobacterium<br>195. g_Zunongwangia<br>196. g_Candidatus_Saccharibacteria_noname<br>197. g_Candidatus_Saccharibacteria_noname_unclassified

		198. g_Chlorobium<br>199. g_Deinococcus<br>200. g_Meiothermus<br>201. g_Methanocaldococcaceae_unclassified<br>202. g_Acidobacteriaceae_unclassified<br>203. g_Granulicella<br>204. g_Cetobacterium<br>205. g_Fusobacterium<br>206. g_Leptotrichia<br>207. g_Leptotrichiaceae_unclassified<br>208. g_Rhodopirellula

Supplementary Table 7: 95% confidence intervals obtained for the mean AUC values for 10 times 10-fold cross validation on the training set for the (a) T2D study (b) Cirrhosis study, (c) Le Chatelier study (Obesity study)

Methods	T2D Mean AUC [95% C.I.]	Cirrhosis Mean AUC [95% C.I.]	Obesity Mean AUC [95% C.I.]
**taxoNN_corr**	**0.832 [0.82, 0.849]**	**0.764 [0.752, 0.781]**	**0.842 [0.83, 0.859]**
RF	0.753 [0.741, 0.77]	0.725 [0.713, 0.742]	0.801 [0.789, 0.818]
SVM	0.731 [0.719, 0.748]	0.717 [0.705, 0.734]	0.751 [0.739, 0.768]
Lasso	0.701 [0.689, 0.718]	0.691 [0.679, 0.708]	0.714 [0.702, 0.731]
Ridge	0.731 [0.719, 0.748]	0.709 [0.697, 0.726]	0.745 [0.733, 0.762]
GBC	0.697 [0.685, 0.714]	0.666 [0.654, 0.683]	0.715 [0.703, 0.732]
NB	0.715 [0.703, 0.732]	0.694 [0.682, 0.711]	0.736 [0.724, 0.753]

Supplementary Table 8: AUC values obtained for both T2D and Cirrhosis studies with varying numbers of generated cases and controls. The last three rows illustrate imbalanced ratios of cases to controls (ranging from 1:3 to 3:4), demonstrating the method's robustness in handling class imbalance. The best AUC is highlighted in bold.

No. of Cases	No. of Controls	AUC on T2D study	AUC on Cirrhosis study
200	200	0.740	0.923
300	300	0.756	0.934
400	400	0.768	0.941
500	500	0.785	0.945
600	600	**0.812**	**0.952**
700	700	0.812	0.952
200	600	0.804	0.941
300	600	0.809	0.947
450	600	0.810	0.948

2 Supplementary Figures

Relative abundance percentage of phyla in the T2D dataset

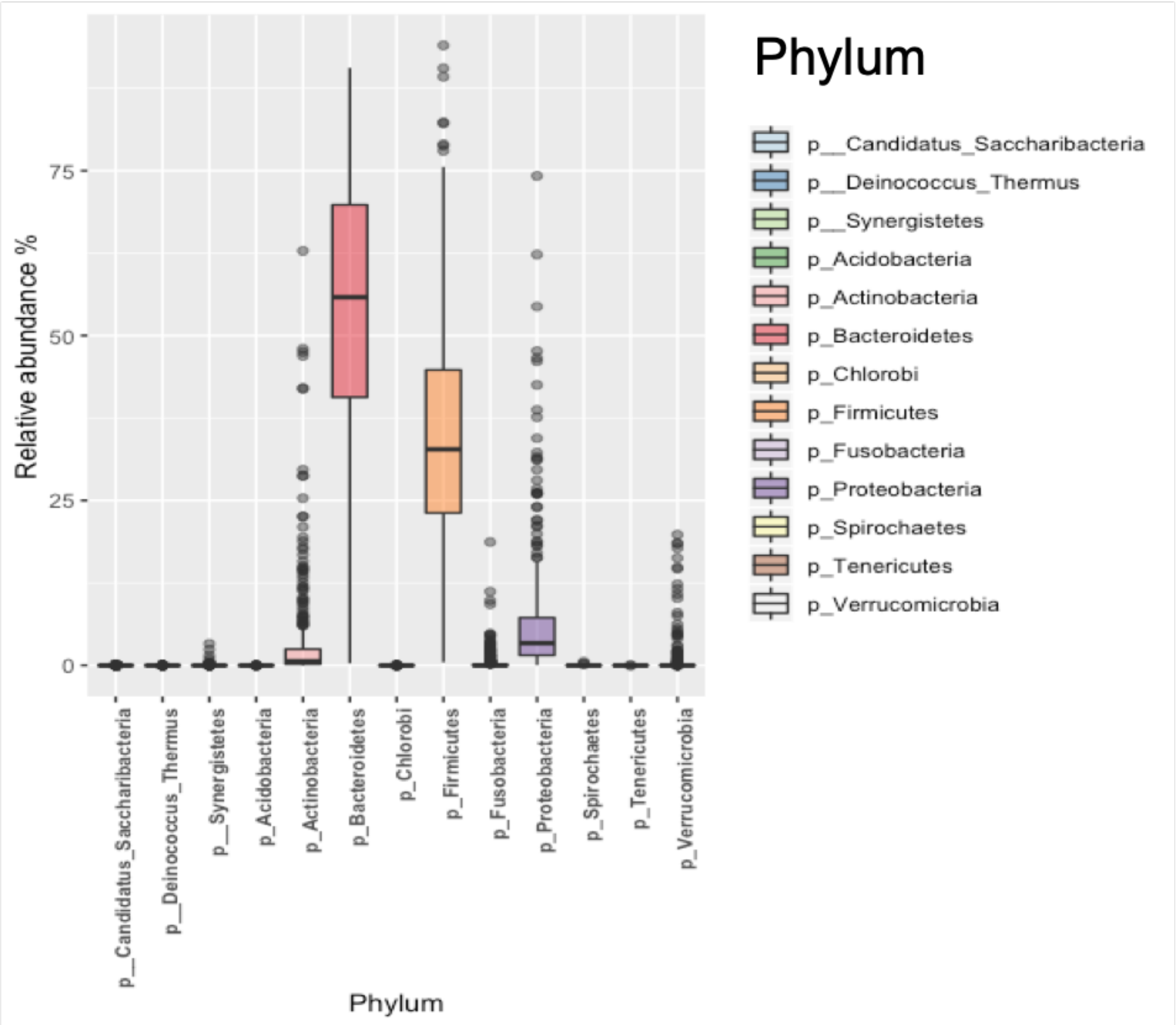

Supplementary Figure 1: Boxplot illustrating relative abundance percentage of OTUs in each phylum of the T2D study. The upper whisker extends from the hinge to the largest value no further than 1.5 * IQR from the hinge (where IQR is the inter-quartile range, or distance between the first and third quartiles). The lower whisker extends from the hinge to the smallest value at most 1.5 * IQR of the hinge. Data beyond the end of the whiskers are called ”outlying” points and are plotted individually.

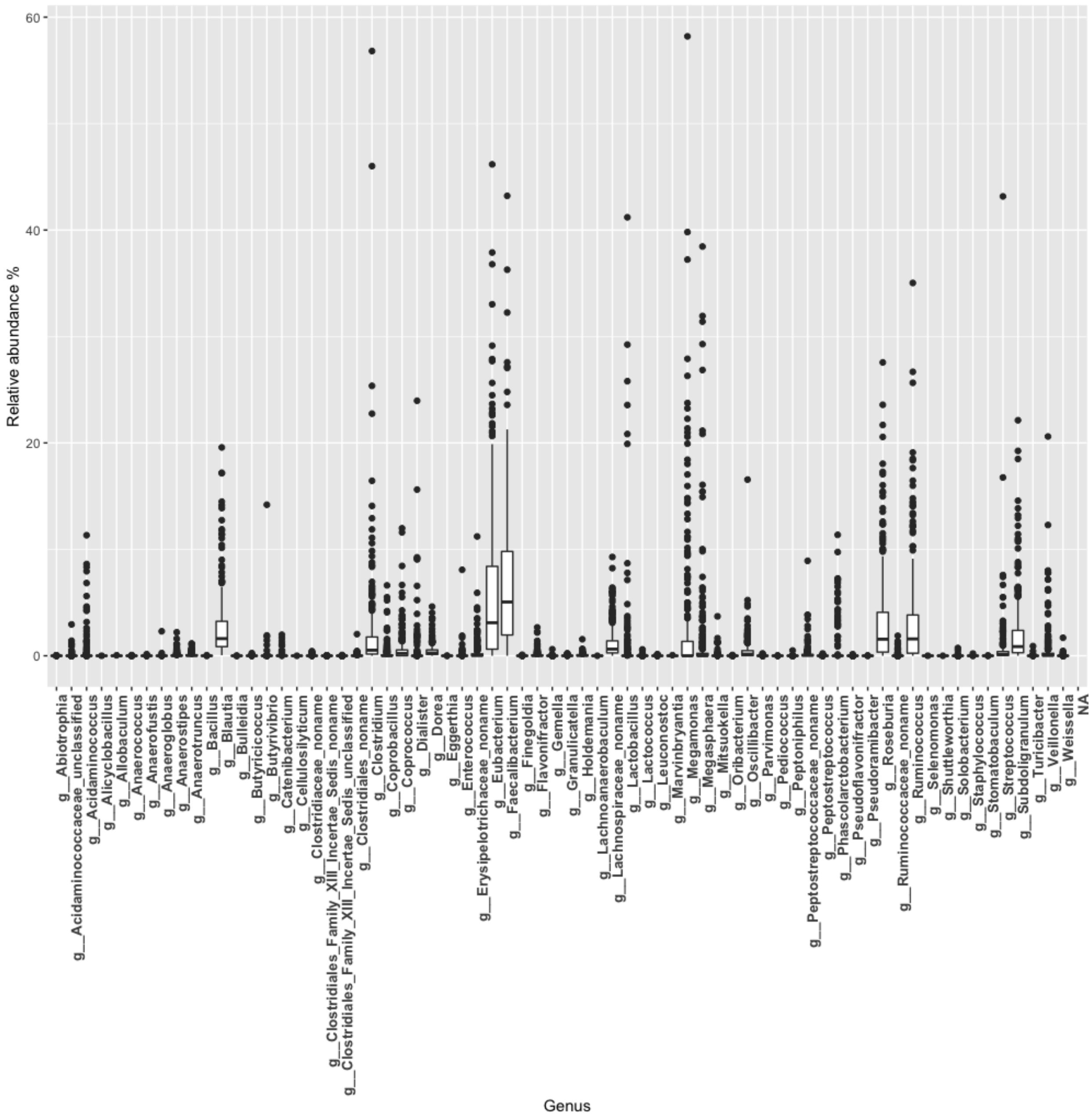

Supplementary Figure 2: Relative abundance percentage of OTUs at genus level in the Firmicutes phylum of the T2D study

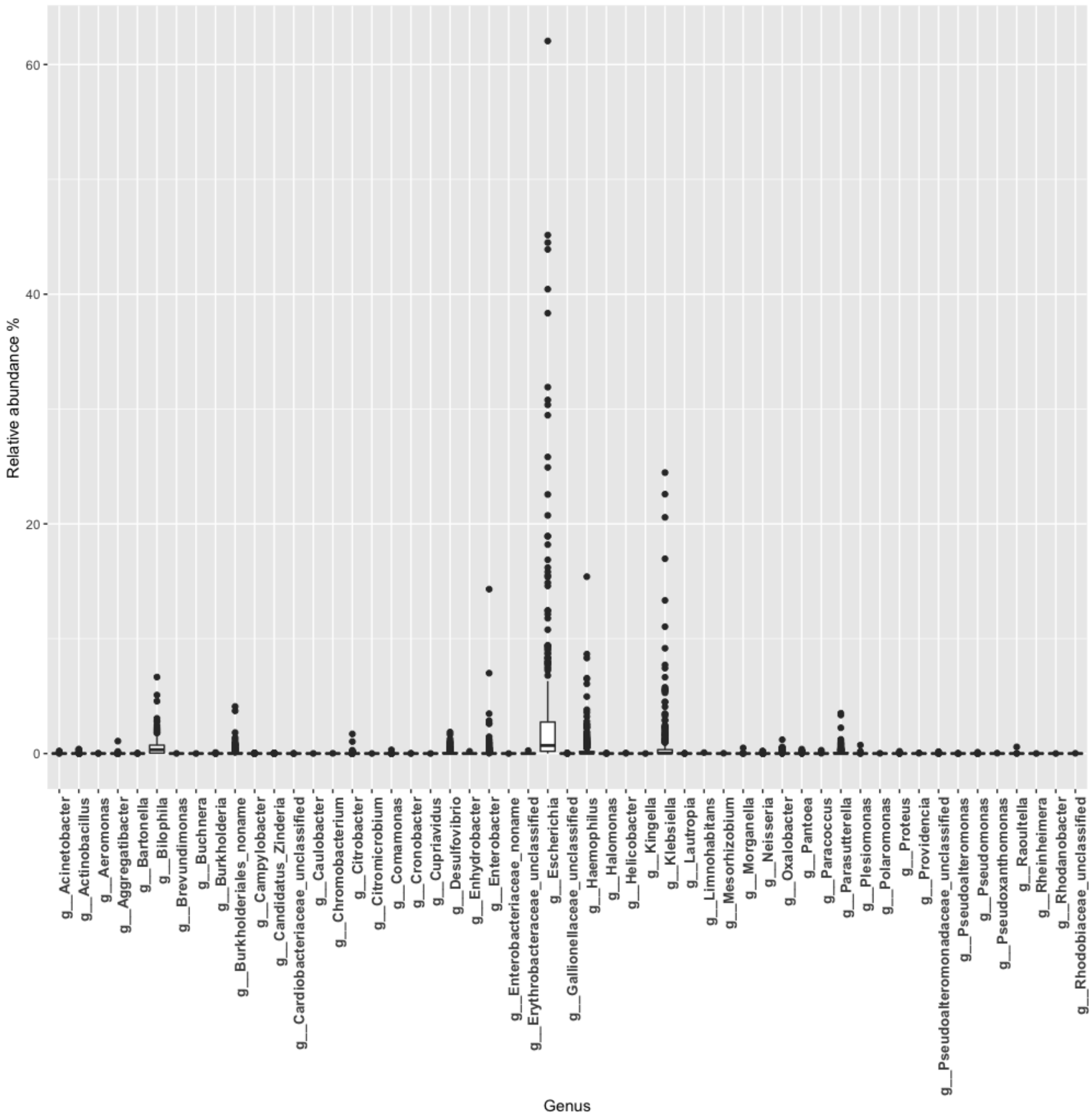

Supplementary Figure 3: Relative abundance percentage of OTUs at genus level in the Proteobacteria phylum of the T2D study

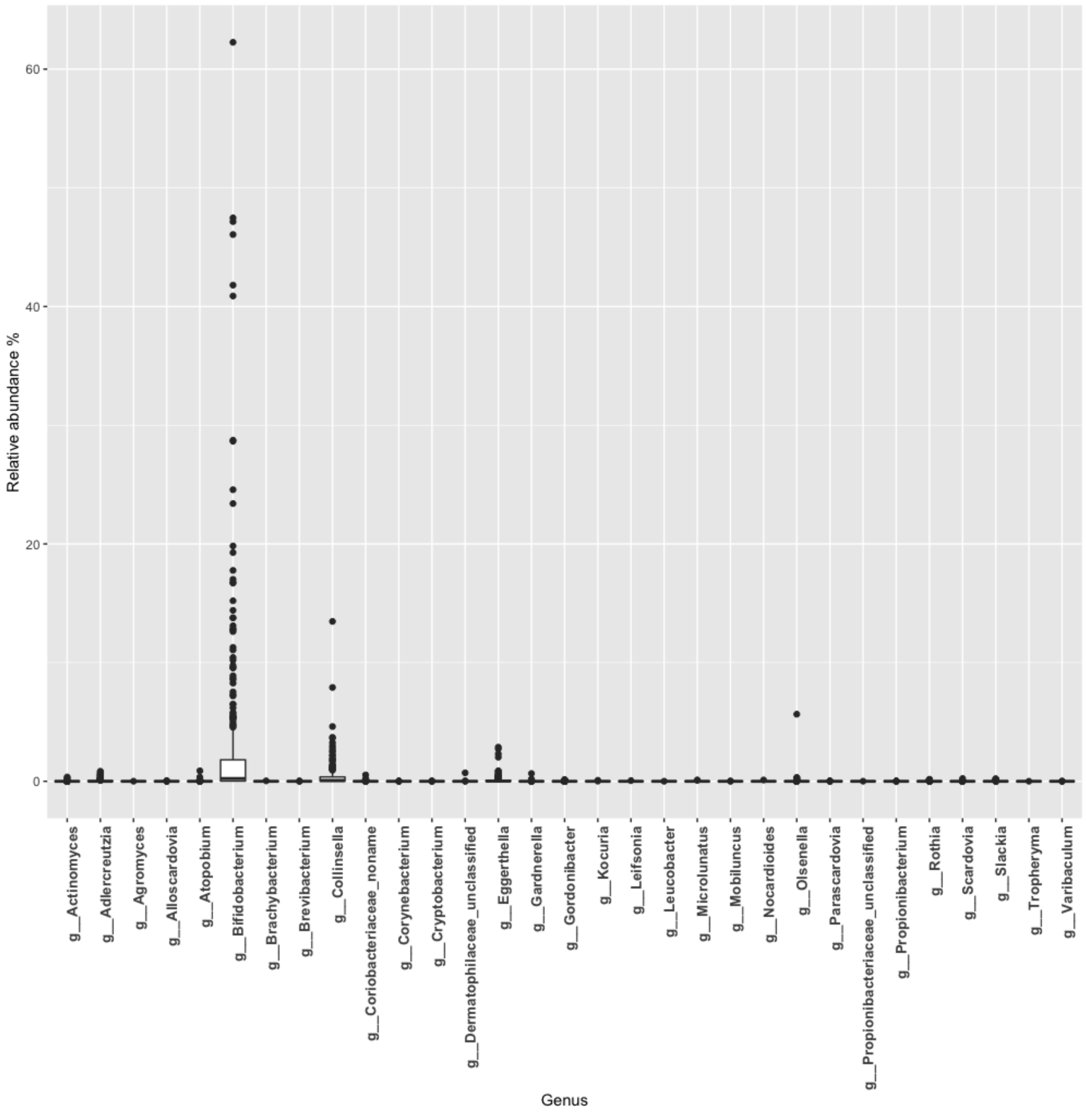

Supplementary Figure 4: Relative abundance percentage of OTUs at genus level in the Actinobacteria phylum of the T2D study

Relative abundance percentage of phyla in the Cirrhosis dataset

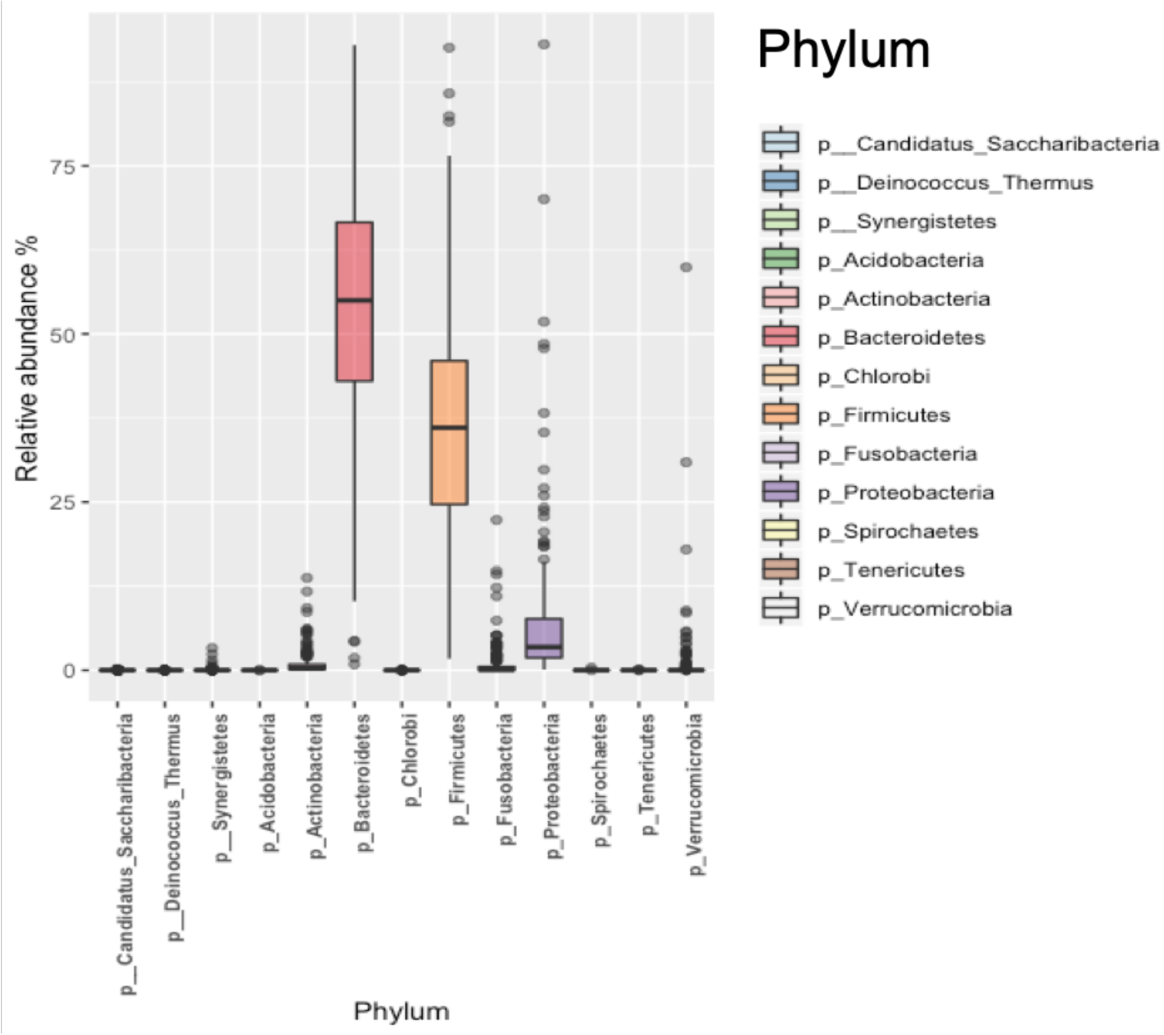

Supplementary Figure 5: Boxplot illustrating relative abundance percentage of OTUs in each phylum of the Cirrhosis study. The upper whisker extends from the hinge to the largest value no further than 1.5 * IQR from the hinge (where IQR is the inter-quartile range, or distance between the first and third quartiles). The lower whisker extends from the hinge to the smallest value at most 1.5 * IQR of the hinge. Data beyond the end of the whiskers are called ”outlying” points and are plotted individually.

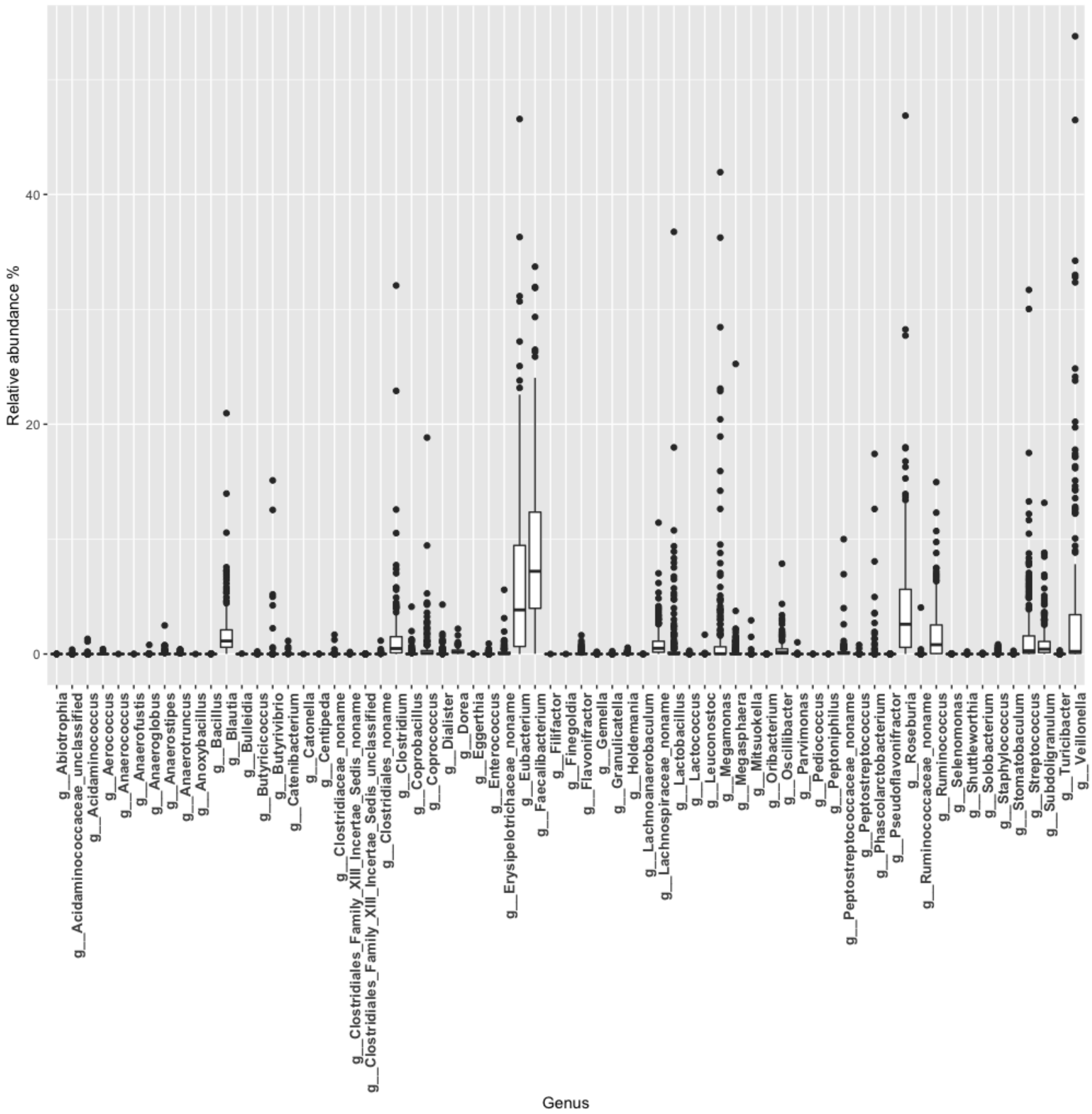

Supplementary Figure 6: Relative abundance percentage of OTUs at genus level in the Firmicutes phylum of the Cirrhosis study

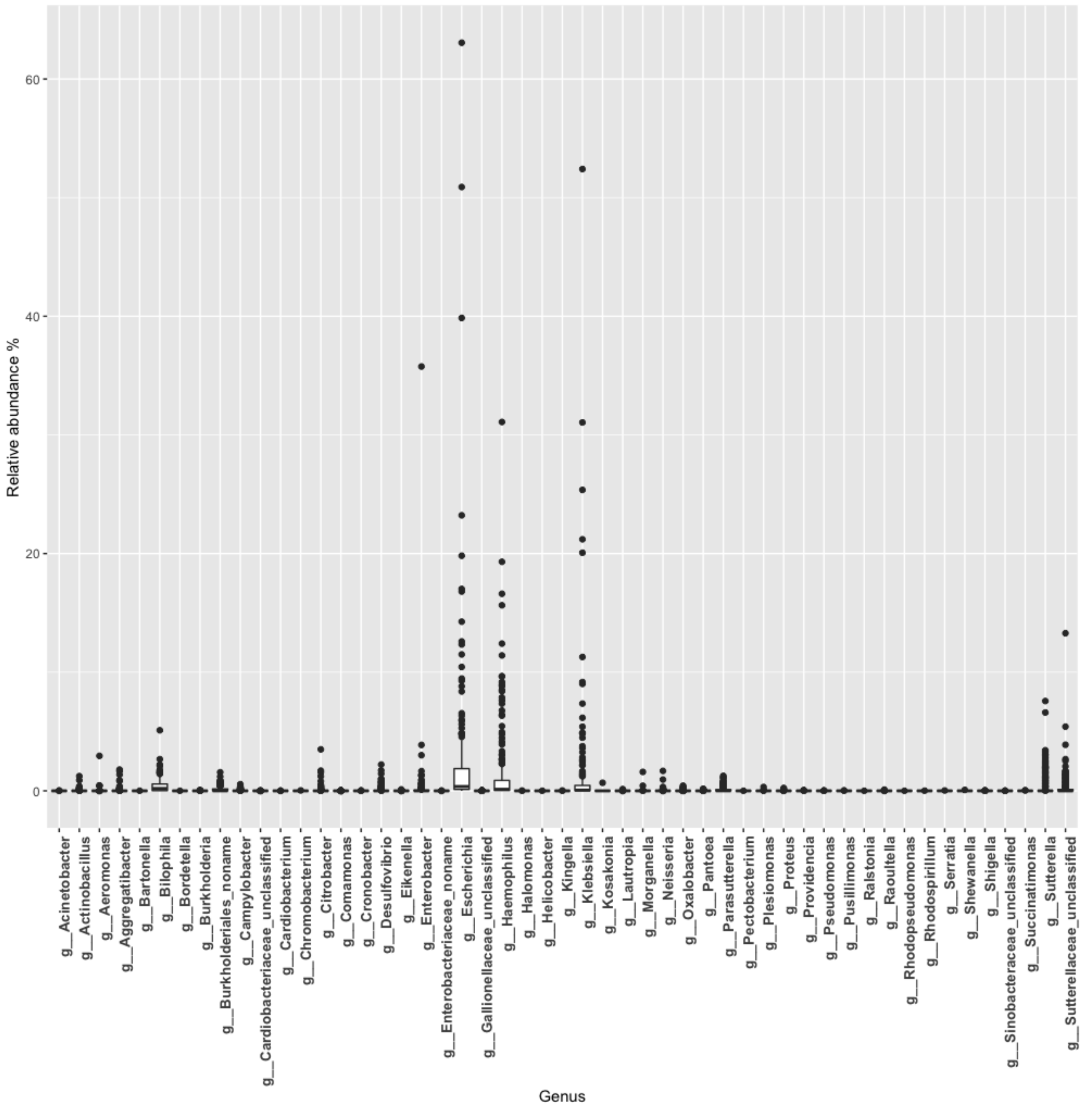

Supplementary Figure 7: Relative abundance percentage of OTUs at genus level in the Proteobacteria phylum of the Cirrhosis study

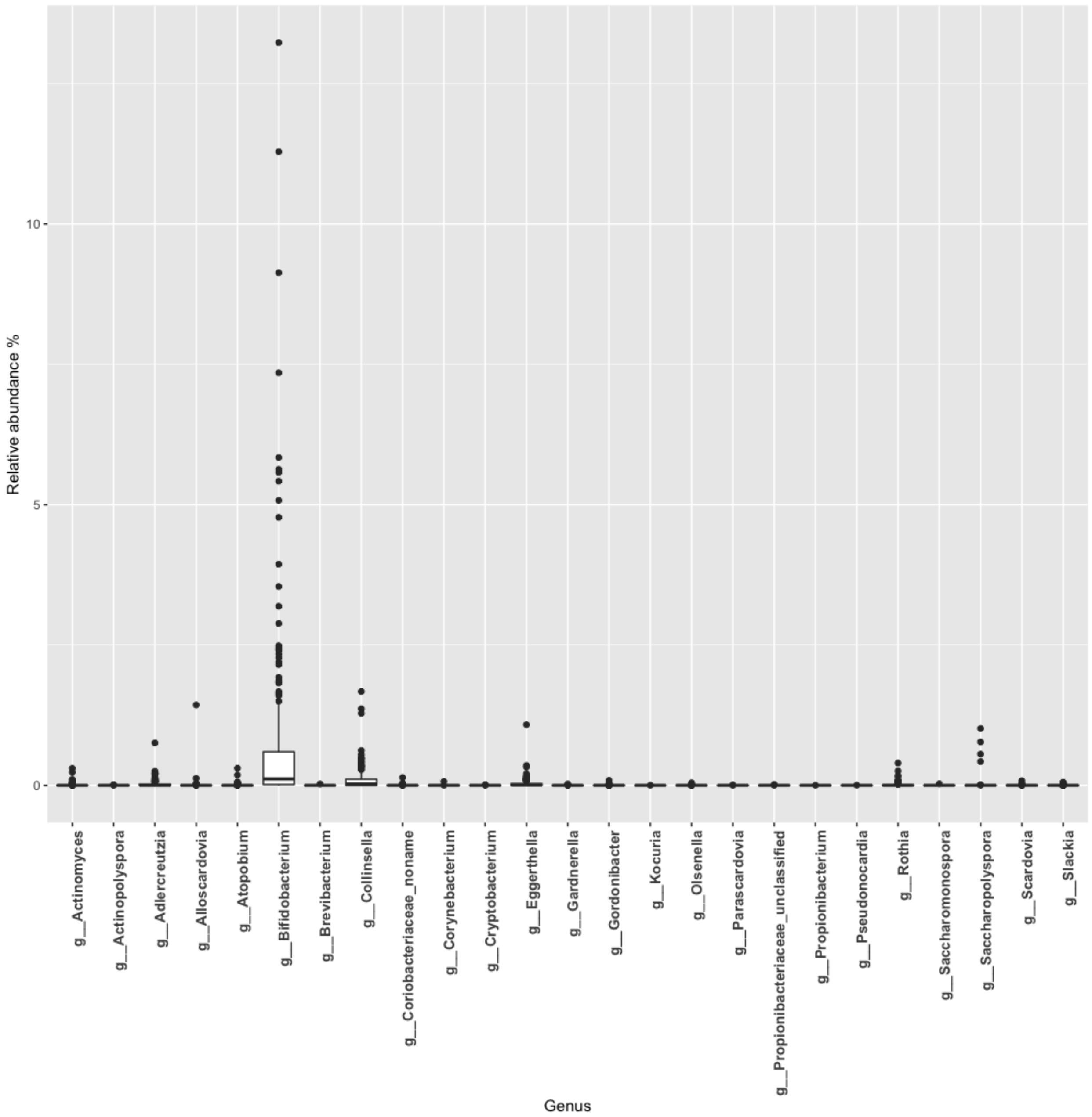

Supplementary Figure 8: Relative abundance percentage of OTUs at genus level in the Actinobacteria phylum of the Cirrhosis study

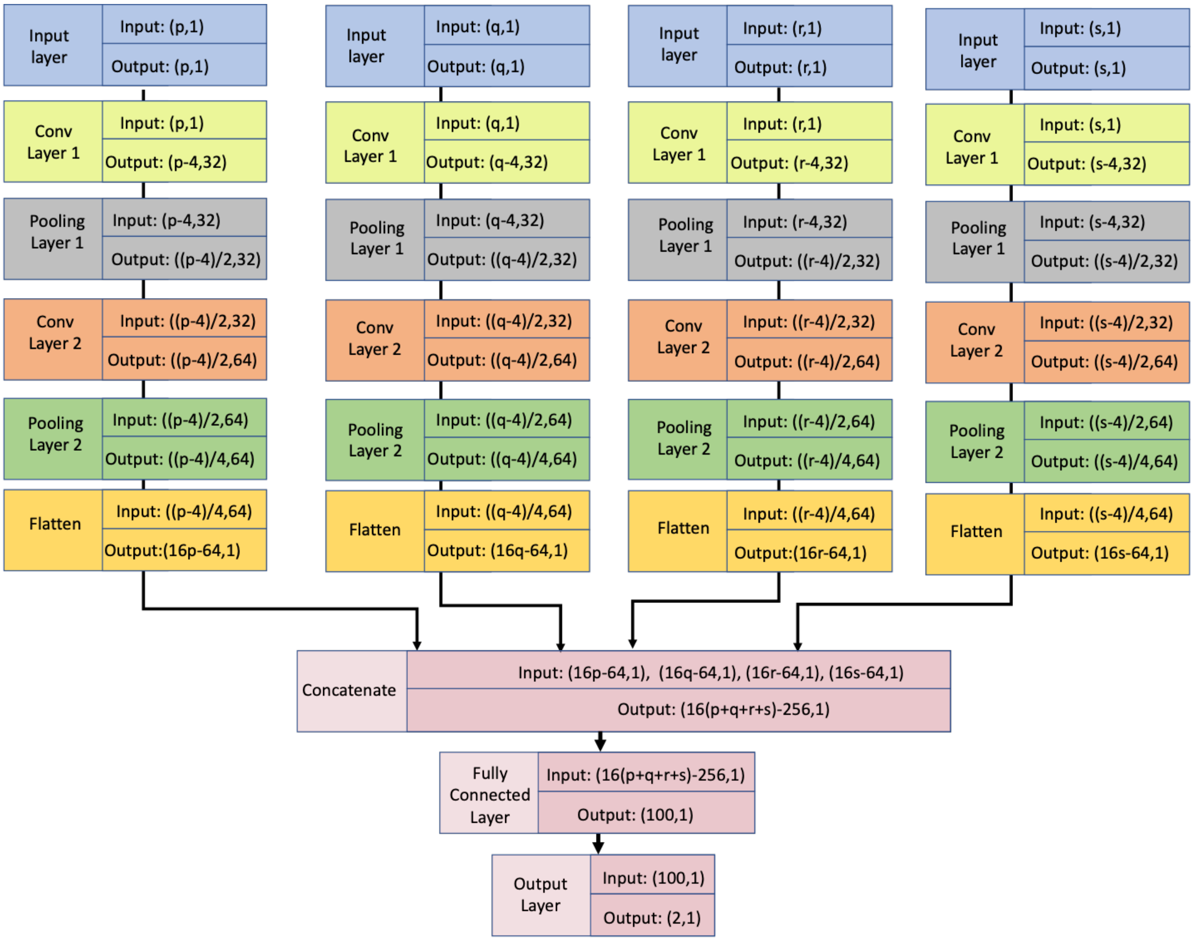

Supplementary Figure 9: Functional working of the layers of $taxoNN$ on 4 clusters of an example dataset containing 'p', 'q', 'r' and 's' OTUs in the respective clusters (where p+q+r+s = N). Each block corresponds to a layer acting on the cluster. Input signifies the dimension of the input to the layer. The input at each step is represented as (k,l) where, 'k' is the number of rows in the input and 'l' represents the number of columns. As the initial input was a vector therefore, l in this case was '1'. Output signifies the dimension of the result after certain operations in that particular layer. Further, as the number of filters increases from 32 in the first Conv layer to 64 in the second Conv layer, the number of columns in the nodes vary from 32 to 64. Finally, in the concatenation step we obtain a single column concatenation vector by stacking flattened vectors from all clusters together.

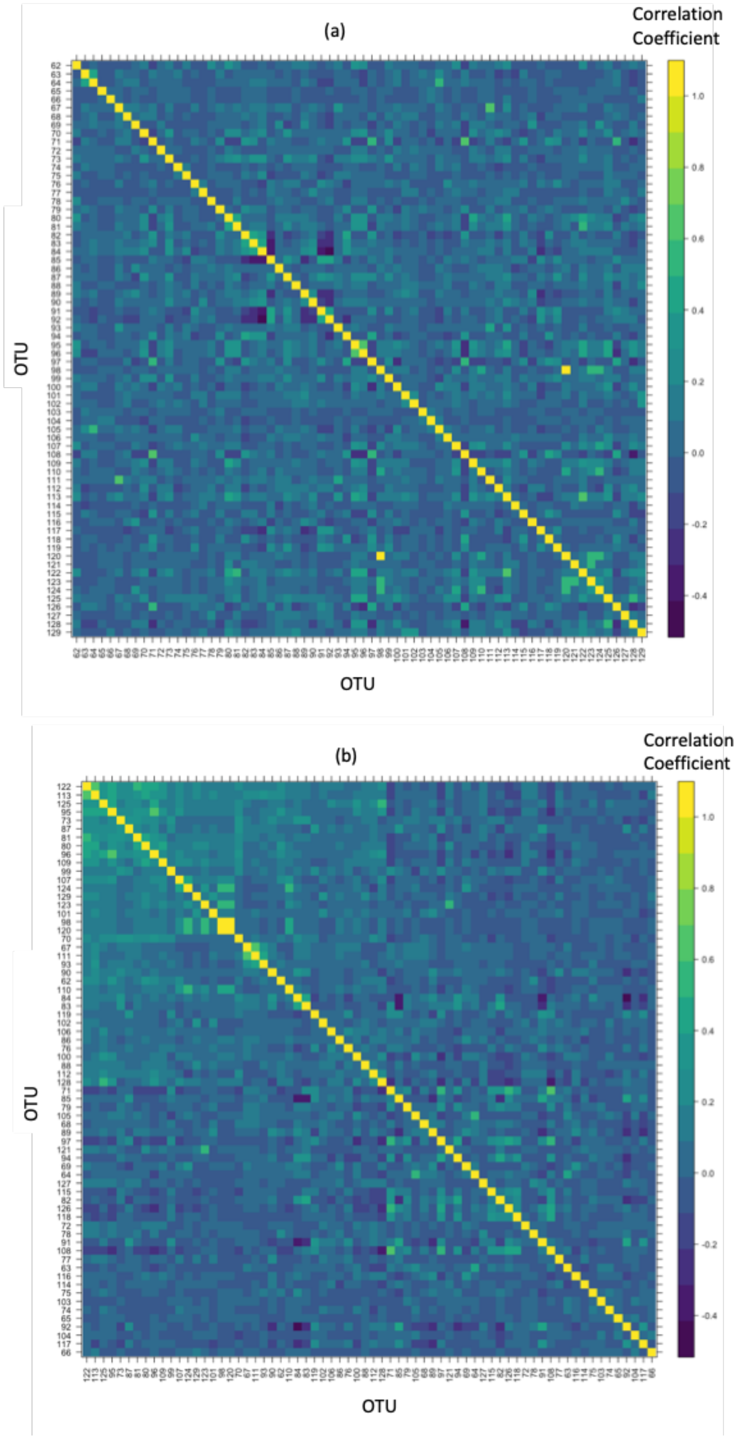

Supplementary Figure 10: Heatmaps for the Spearman rank of the OTUs in the cluster, Phylum Firmicutes, (a) before ordering and (b) after the ordering based on correlation of the OTUs in the T2D study

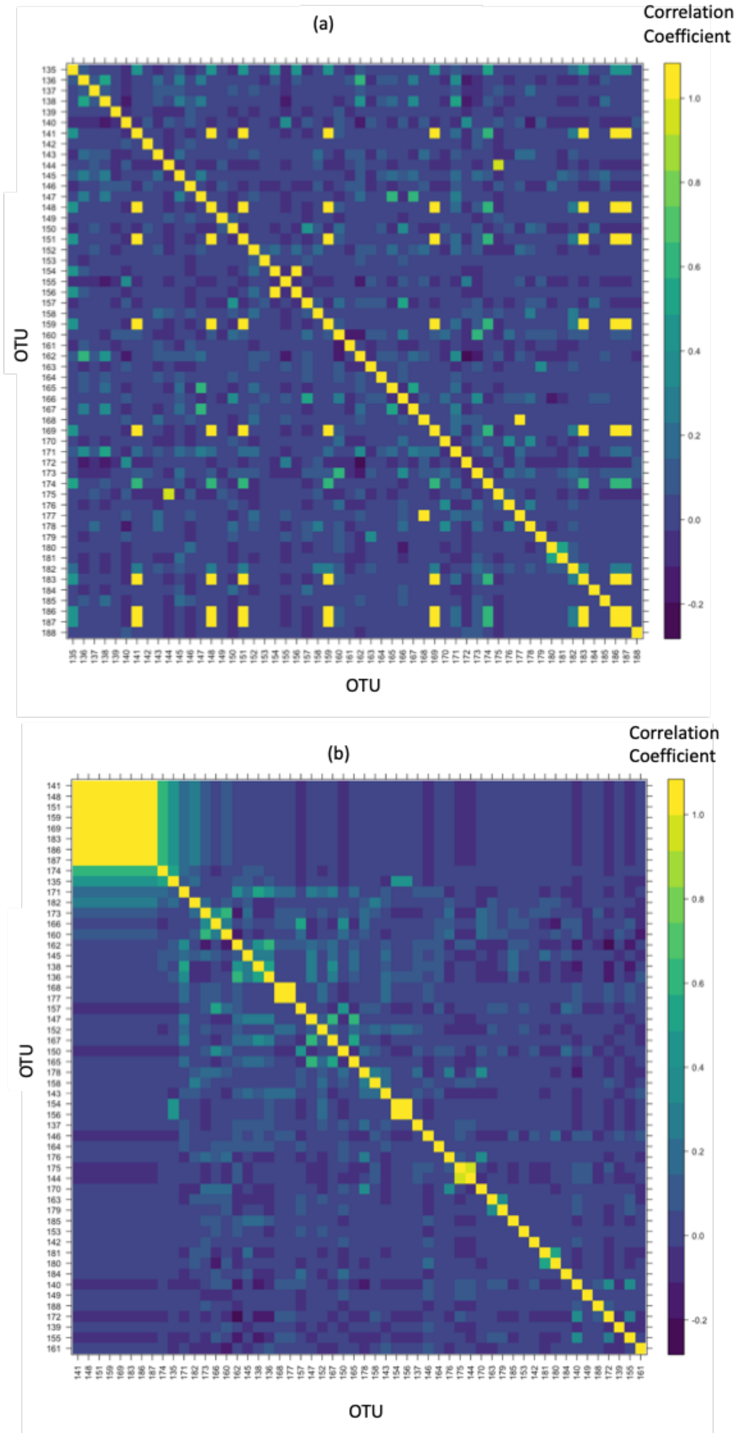

Supplementary Figure 11: Heatmaps for the Spearman rank of the OTUs in the cluster, Phylum Proteobacteria, (a) before ordering and (b) after the ordering based on correlation of the OTUs in the T2D study

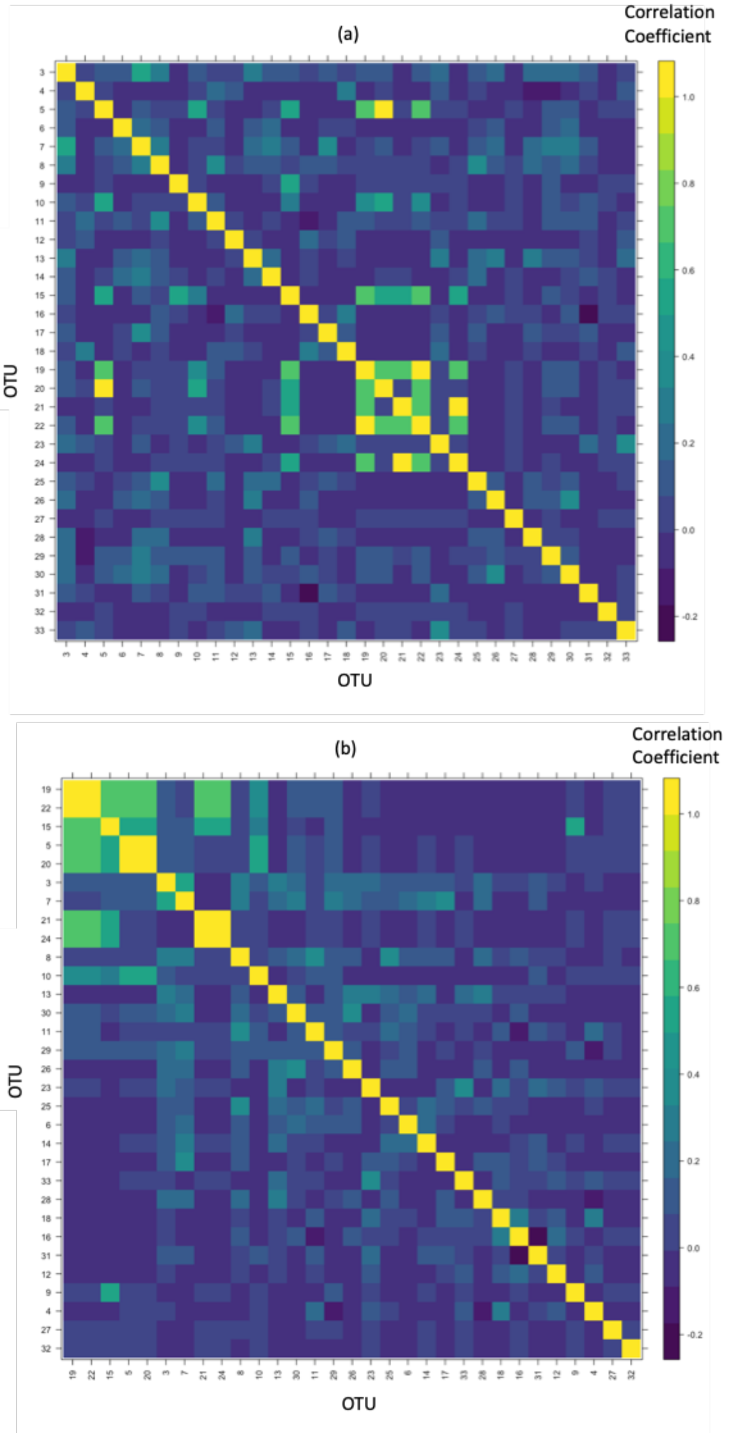

Supplementary Figure 12: Heatmaps for the Spearman rank of the OTUs in the cluster, Phylum Actinobacteria, (a) before ordering and (b) after the ordering based on correlation of the OTUs in the T2D study

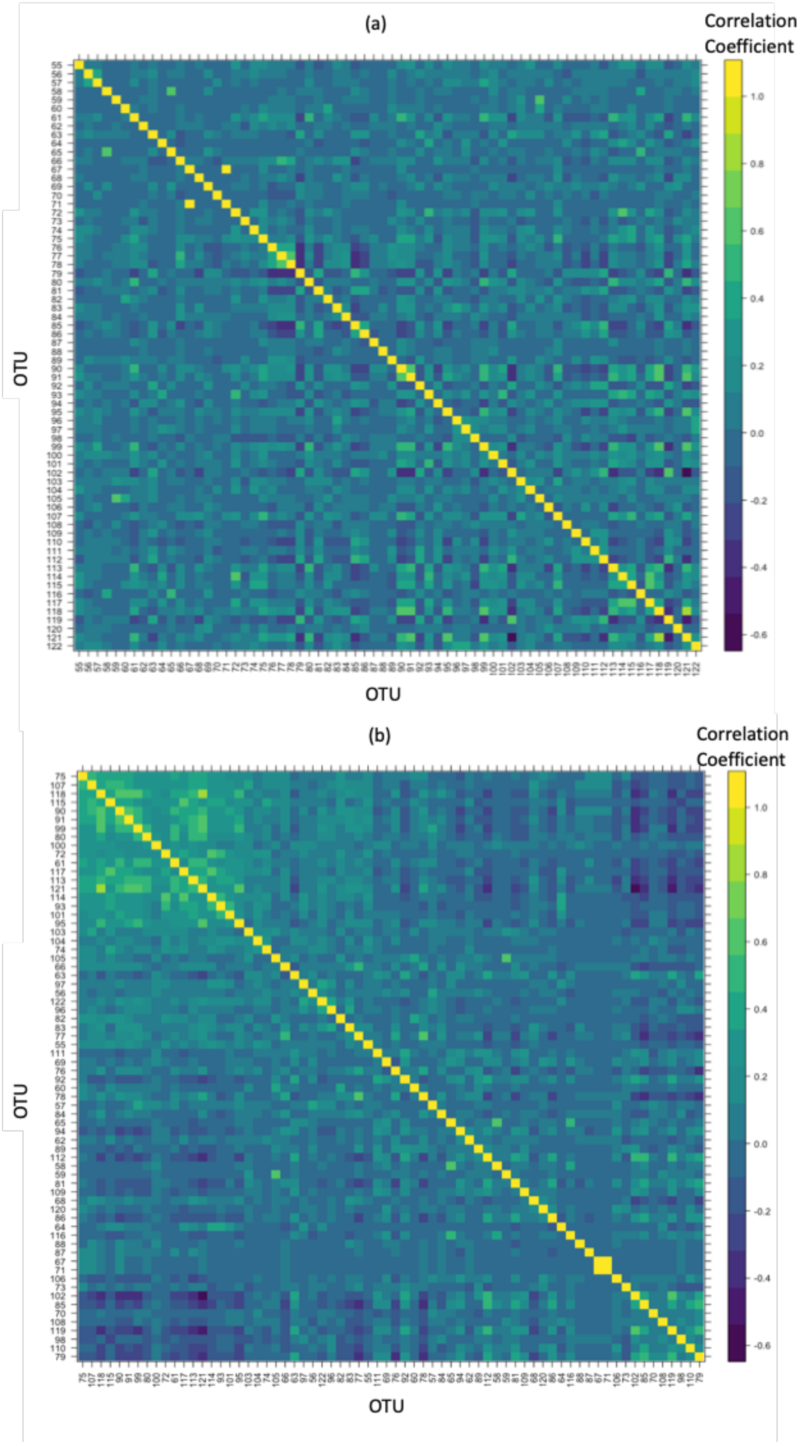

Supplementary Figure 13: Heatmaps for the Spearman rank of the OTUs in the cluster, Phylum Firmicutes, (a) before ordering and (b) after the ordering based on correlation correlation of the OTUs in the Cirrhosis study

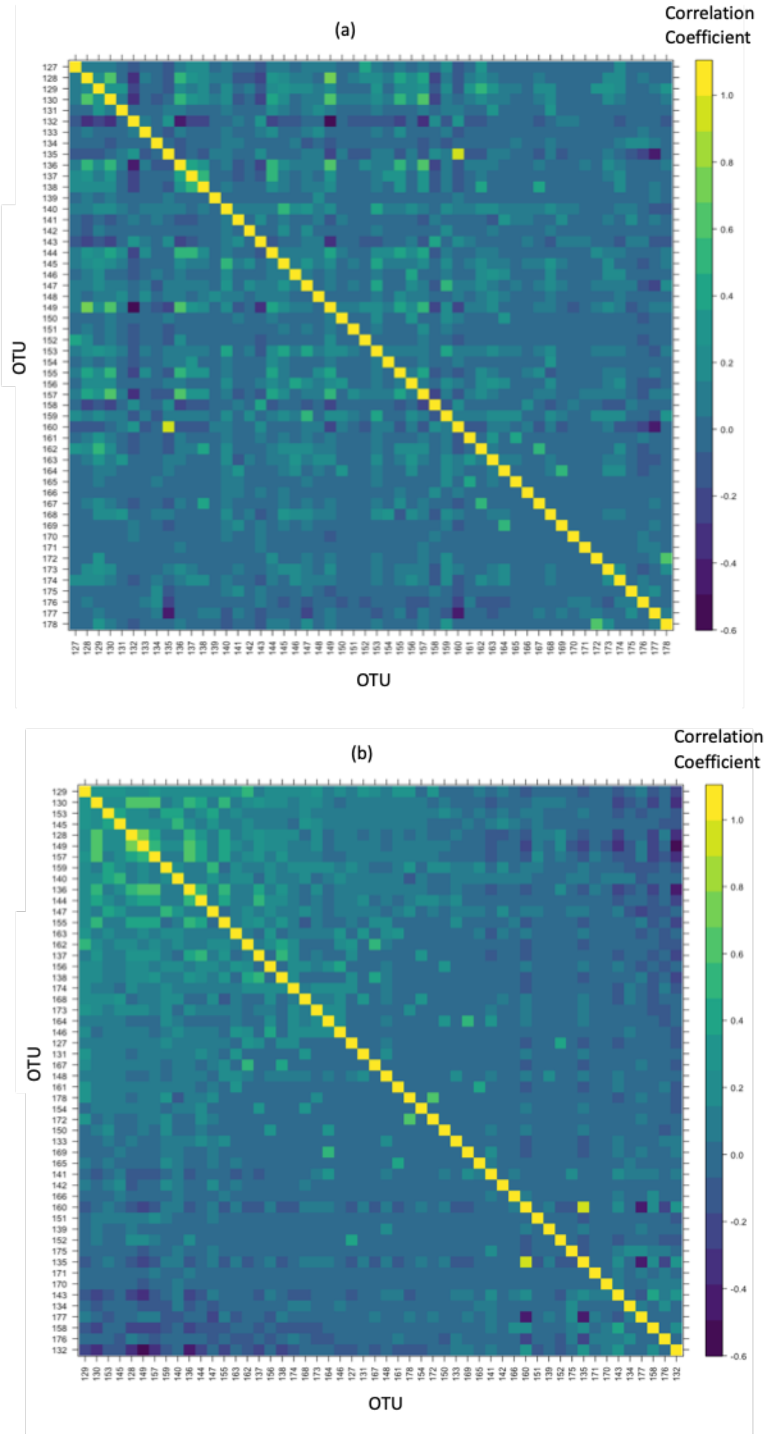

Supplementary Figure 14: Heatmaps for the Spearman rank of the OTUs in the cluster, Phylum Proteobacteria, (a) before ordering and (b) after the ordering based on correlation correlation of the OTUs in the Cirrhosis study

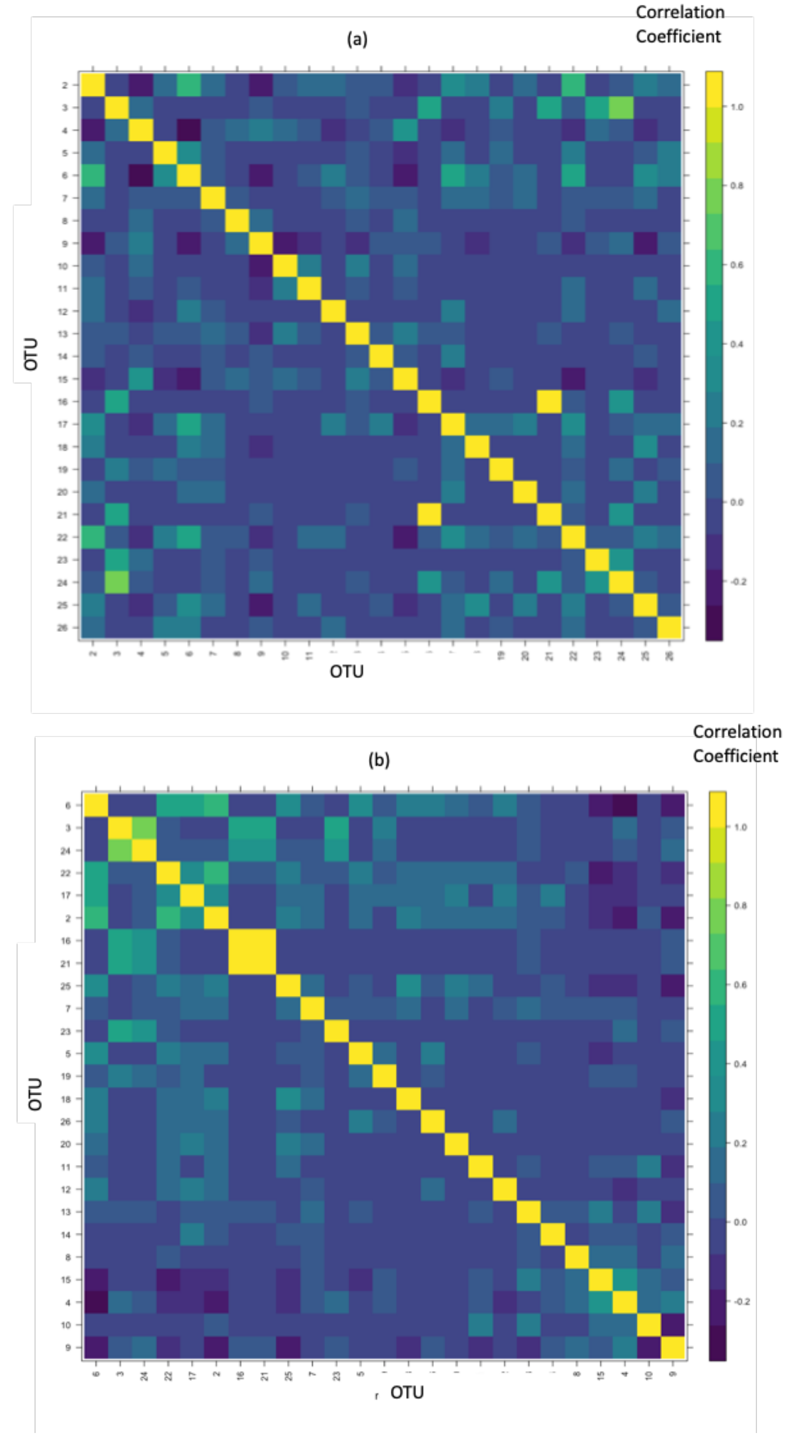

Supplementary Figure 15: Heatmaps for the Spearman rank of the OTUs in the cluster, Phylum Actinobacteria, (a) before ordering and (b) after the ordering based on correlation correlation of the OTUs in the Cirrhosis study

3 Supplementary Methods

3.1 Extracting important features through Integrated Gradient Methodology

To identify the important variables in our predictive modeling, we used the Integrated Gradient (IG) methodology [4] which is an interpretability technique for deep neural networks. IG helps in visualizing the input variable importance that contributes to the model's prediction. We calculated gradients to measure the relationship between changes to a variable and corresponding changes in the model's predictions. The gradient informs which variable has the strongest effect on the models predicted class probabilities. Varying the input variable changes the output, and hence each input feature will receive some attribution during the interpolation which in turn helps to calculate the variable importance for the input. A variable that does not affect the output gets no attribution. Finally, the numerical approximation through averaging the gradients is computed. The higher the gradient, the more important the feature is considered to the classification task.

Supplementary Figure 16: Identifying variable importance through IG approach in NN modeling. (a) Top-10 important OTUs at the genus level for predicting disease status in the Cirrhosis study and (b) Top-10 important OTUs at the genus level for predicting disease status in the T2D study. The higher the gradient more important the feature.

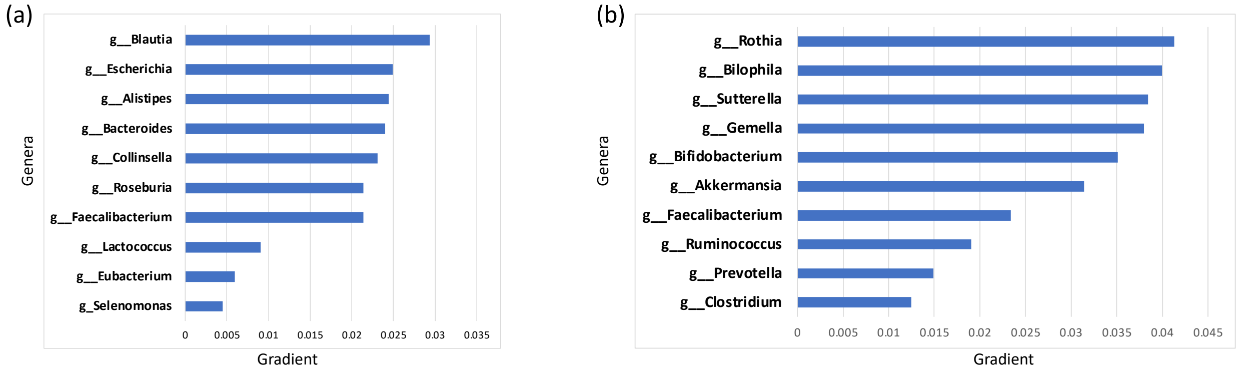

4 References

[1] Qin, J. *et al.* A metagenome-wide association study of gut microbiota in type 2 diabetes. *Nature* **490**, 55–60 (2012).

[2] Qin, N. *et al.* Alterations of the human gut microbiome in liver cirrhosis. *Nature* **513**, 59–64 (2014).

[3] Le Chatelier, E. *et al.* Richness of human gut microbiome correlates with metabolic markers. *Nature* **500**, 541–546 (2013).

[4] Sundararajan, M., Taly, A. & Yan, Q. Axiomatic attribution for deep networks. In *International conference on machine learning*, 3319–3328 (PMLR, 2017).
